# Rapid increase in snake dietary diversity and complexity following the end-Cretaceous mass extinction

**DOI:** 10.1371/journal.pbio.3001414

**Published:** 2021-10-14

**Authors:** Michael C. Grundler, Daniel L. Rabosky

**Affiliations:** Museum of Zoology and Department of Ecology and Evolutionary Biology, University of Michigan, Ann Arbor, Michigan, United States of America; Universidade de São Paulo, BRAZIL

## Abstract

The Cenozoic marked a period of dramatic ecological opportunity in Earth history due to the extinction of non-avian dinosaurs as well as to long-term physiographic changes that created new biogeographic theaters and new habitats. Snakes underwent massive ecological diversification during this period, repeatedly evolving novel dietary adaptations and prey preferences. The evolutionary tempo and mode of these trophic ecological changes remain virtually unknown, especially compared with co-radiating lineages of birds and mammals that are simultaneously predators and prey of snakes. Here, we assemble a dataset on snake diets (34,060 observations on the diets of 882 species) to investigate the history and dynamics of the multidimensional trophic niche during the global radiation of snakes. Our results show that per-lineage dietary niche breadths remained remarkably constant even as snakes diversified to occupy disparate outposts of dietary ecospace. Rapid increases in dietary diversity and complexity occurred in the early Cenozoic, and the overall rate of ecospace expansion has slowed through time, suggesting a potential response to ecological opportunity in the wake of the end-Cretaceous mass extinction. Explosive bursts of trophic innovation followed colonization of the Nearctic and Neotropical realms by a group of snakes that today comprises a majority of living snake diversity. Our results indicate that repeated transformational shifts in dietary ecology are important drivers of adaptive radiation in snakes and provide a framework for analyzing and visualizing the evolution of complex ecological phenotypes on phylogenetic trees.

## Abstract 2

The Cenozoic marked a period of dramatic ecological opportunity in Earth history due to the extinction of non-avian dinosaurs and long-term physiographic changes. This phylogenetic natural history study offers new insights into the evolution of snake ecological diversity after the end-Cretaceous mass extinction, as they took advantage of these new opportunities.

## Introduction

Evolutionary divergence in feeding ecology is a fundamental response to both ecological opportunity and interspecific competition, often involving coordinated change in prey preferences, foraging habitat, and trophic morphology [1,2]. The origin of new feeding modes is a defining characteristic of many adaptive radiations, including such well-known examples as cichlid fishes and Hawaiian honeycreepers [3]. Ecological release from antagonistic effects of competition and predation leads to the expectation that lineages will quickly diverge in response to ecological opportunity, resulting in “burst-like” dynamics, whereby niche divergence evolves rapidly early in the history of a diversifying clade and gradually slows as lineages evolve into new ecological modalities and saturate accessible ecospace [4–6]. This process may repeat itself as new opportunities arise in the form of biotic turnover after extinction, dispersal to new biogeographic theaters, or the origin of novel phenotypes that alter how an organism interacts with its environment. As a result, the history of many clades can be described by a series of transformational shifts on an ever-changing adaptive landscape [7,8].

The end-Cretaceous extinction event marked the beginning of a dramatic period of ecological opportunity in Earth history. The extinction of non-avian dinosaurs and the resulting availability of uncontested ecospace set the stage for spectacular inter- and intra-ordinal diversification of birds and mammals in the early Cenozoic [9–15]. Continental tectonics and long-term climate cooling throughout the Cenozoic created further ecological opportunity in the form of new biogeographic theaters (e.g., the separation of Australia from Antarctica) and new habitats (e.g., the spread of grasslands) [16–18]. The massive ecological diversification of birds and mammals in response to these opportunities reshaped ecological communities of both land and sea, and the origin of new trophic modalities was a key part of this process [19–21]. So impressive was the diversification of mammals that the Cenozoic is commonly referred to as the “Age of Mammals.”

With nearly as many species of snakes as there are mammals, however, the Cenozoic might just as well be called the “Age of Snakes” [22]. Numbering almost 4,000 species—the vast majority of which diversified in the wake of the K-Pg extinction (S1 Fig) [23,24]—snakes comprise a global radiation that accounts for over 10% of terrestrial vertebrate diversity. Snake evolution has given rise to an enormous variety of feeding habits, many of which are highly specialized and substantially different from the diets of other squamate reptiles (lizards). Numerous functional innovations facilitated the evolutionary expansion of snake diets, including the origin of novel prey subjugation behaviors [25,26], highly kinetic skulls with complex musculature [27,28], and sophisticated venom delivery systems [29–32]. The Cretaceous ancestors of modern-day snakes were already ecologically diverse [33–36], but the massive ecological shifts during the period of snake diversification following the K-Pg extinction are poorly characterized. It was during this time that snake communities familiar to present-day observers were forming, and a better understanding of the trophic transformations that took place will help inform hypotheses regarding links between snake dietary adaptations and lineage diversification [28,30,37,38].

The dietary specialization observed in most snakes combined with their high diversity further suggests that knowledge of tempo and mode in snake diet evolution may also yield more general insights into the mechanisms by which ecological and morphological novelty arises in adaptively radiating clades. The preponderance of diverse clades of dietary specialists among snakes [39] suggests, for example, that ecological specialists are no less evolutionarily versatile (sensu [40]) than ecological generalists, perhaps pointing to the importance of behavioral flexibility or a labile feeding apparatus in facilitating ecological shifts [41–43]. Snakes also display a complex mixture of generalized and specialized morphologies related to diet, and understanding the pattern and timing of ecological shifts in relation to phenotype may help answer questions about the roles of ecological opportunity and developmental constraints as controls on adaptive radiation [44,45].

**Fig 1:**
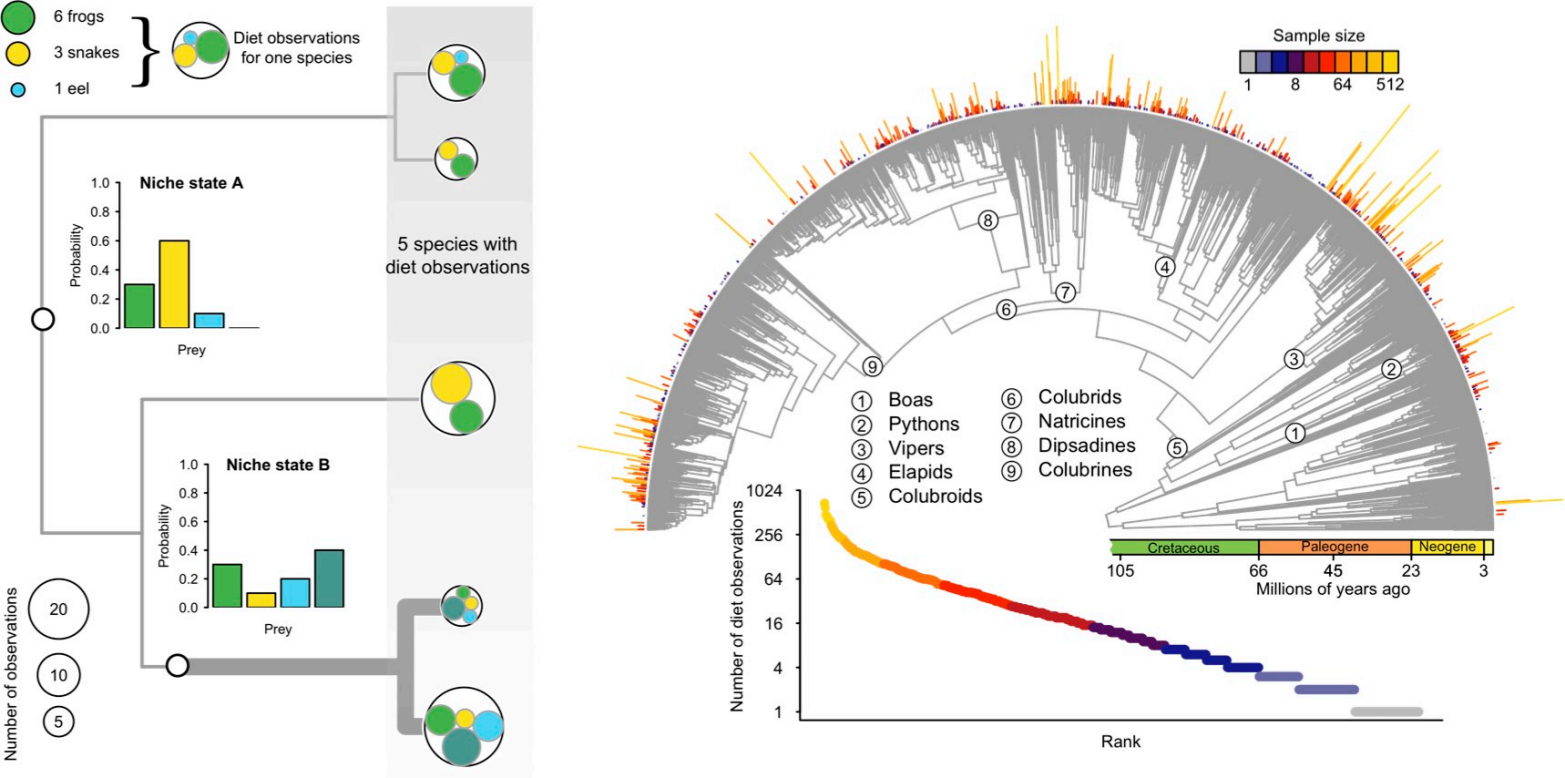
**Inference model (left) and empirical dataset (right) used for the reconstruction of multivariate ecological phenotypes on phylogenetic trees. Left:** Hypothetical sample data illustrating the use of primary natural history data for estimating dietary niches. Observed data are the sets of counts of different prey items recorded in the sampled diets of 5 hypothetical species. Subcircles (green, yellow, and blue) denote the number of observations of different food resources within each of the 5 hypothetical species (open circles). These data represent “primary natural history data” as they originate from direct observations of organisms in nature or from examination of museum specimens. The model we developed assumes these data are generated from a set of latent (unobserved) niche states that correspond to distinct multinomial distributions over a set of prey categories (inset bar plots). The inference framework uses the observed data and phylogeny to infer the set of latent niche states and their phylogenetic distribution. Here, an ancestral niche state (thin branches) underwent a trophic shift to niche state “B” (thick branches), resulting in a paraphyletic assemblage of 3 species that share the ancestral niche state (“A”) and a set of 2 species characterized by the derived niche state (“B”). The derived state in this example is associated with trophic expansion, adding a novel resource (shown in teal) to the proportional prey utilization spectrum. Note that sampled diets for individual species vary, even for those sharing a common niche state, because the “true” niche state is assumed to be a probability distribution and is therefore characterized by intraspecific sampling variability. **Right:** Comprehensive species-level phylogeny of snakes from [47] highlighting major clades, evolutionary timescale, and sample size distribution for number of prey use observations. Rank abundance curve below the phylogeny and segments along the outer semicircle depict the sample size distribution for all snakes with diet observations. Gaps along the outer semicircle occur for species with no diet observations, and these species were pruned from the phylogeny prior to analysis. A total of 34,060 primary natural history observations of prey acquisition by 882 species of snakes were collated for analysis. The data underlying this figure may be found in doi: 10.5281/zenodo.4446064.

In this study, we describe the dynamics of trophic niche evolution across extant snakes, combining multivariate natural history observations and a new modeling framework to investigate evolutionary tempo and mode of the multidimensional trophic niche. We assembled a dataset on snake diets (34,060 primary natural history observations of prey acquisition for 882 species) and used these data together with a new stochastic model–based comparative method that we previously developed to reconstruct evolutionary histories of dietary change from primary natural history data [46]. Our use of primary data means that the analysis method explicitly accounts not only for the multidimensional nature of the trophic niche but also for differences in the amount of data per species, so that uncertainty due to lack of knowledge is integrated into the method (Fig 1).

We find that after an initial shift away from eating invertebrates, the diversity of snake feeding habits increased rapidly after the K-Pg boundary, with substantial increases in the rate of trophic innovation associated with colonization of the Nearctic and Neotropical realms. Our results demonstrate the potential of primary natural history data for broadscale inference in macroevolution and underscore the role of repeated transformational shifts in dietary ecology in driving snake adaptive radiation.

## Results and discussion

The merged phylogenetic and diet dataset contains 882 species representing 356 genera from nearly all snake families (the only exceptions being Anomochilidae and Gerrhopilidae). Per-species sample sizes (number of observed prey items) range from 1 to 746 with a mean of 38 and a median of 12, while per-genus sample sizes range from 1 to 2,753 with a mean of 95 and a median of 25, for a total of 34,060 observations (Fig 1). Most observations in the database are from direct encounters with snakes in the field or from dissections of preserved museum specimens. Combining these 2 sources of data results in a more complete picture of the prey spectrum consumed by any given species, as field and museum specimens sometimes differ in relative frequencies of recorded prey types [48]. Snake diets can vary within species, driven by sexual and ontogenetic differences in body size and by geographic variation in available prey types [49,50]. Our compilation records these details when possible, but the dataset used for analysis in the present study aggregates all records available for a given species, thereby creating a composite picture of the prey spectrum sampled by individual species.

Observational natural history data, especially with regard to snake diets, present analytical challenges because sampling is typically highly uneven across species and because the data are strongly zero inflated. We developed a Bayesian phylogenetic comparative method that models dietary niche states as unobserved multinomial distributions from which observed diet data are sampled [46]. The new method uses phylogeny and the observed counts of sampled prey items to jointly infer continuous dietary niche states for each species and their unsampled ancestors (Methods). The resulting trophic network structure is informed by both the observed diet data and the phylogenetic relationships of sampled species, and these 2 sources of information allow us to incorporate observations from species with highly variable sample sizes because the model can use information from well-sampled phylogenetic relatives to estimate dietary niches for species with poorly characterized diets.

**Fig 2:**
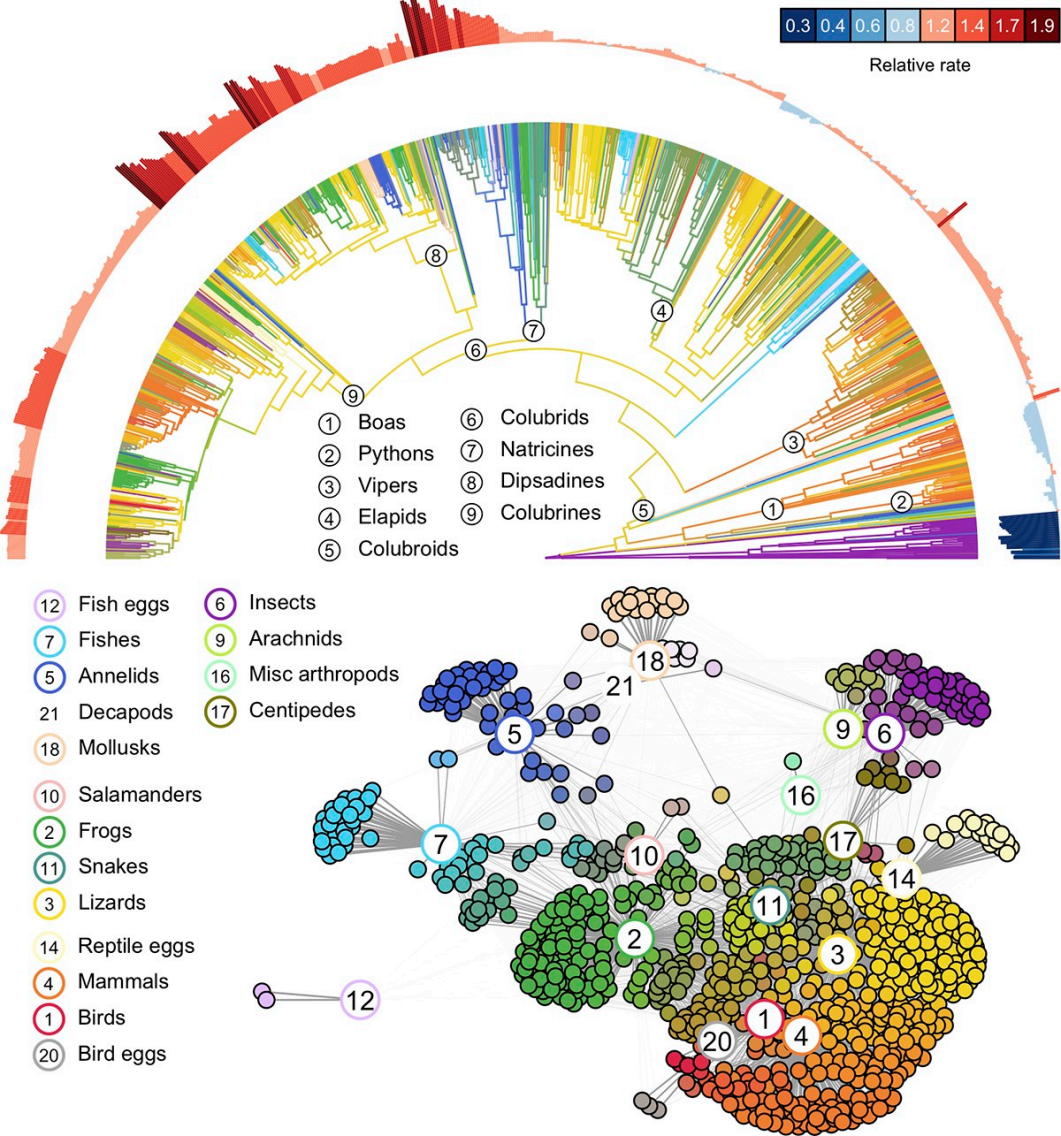
**Evolutionary dynamics of diet evolution across the radiation of extant snakes (top) and model inferred trophic network structure (bottom) estimated from 34,060 primary natural history observations of prey acquisition. Top:** Reconstructions of ancestral snake diets (branch colors) and evolutionary rates of prey switching (outer semicircle) were inferred using a Dirichlet-multinomial Markov model for multivariate ecological trait evolution. Branch colors denote reconstructed patterns of resource use and are colored according to the same scheme used in the bottom panel. Outer semicircle denotes average rates of diet evolution for each taxon expressed relative to the average for all snakes (see text for details). Evolutionary rates are higher for the colubroid mega-radiation, which accounts for the majority of global snake diversity. Despite generally lower evolutionary rates, however, non-colubroid snakes display a similar breadth of feeding modalities as colubroids. Time-calibrated phylogeny for the 882 species for which diet observations were available was taken from [47]. **Bottom:** Graphical network illustrating connections between prey types (numbered circles) and individual snake taxa (filled circles). Lines connect snake taxa to prey items in their diets, and line widths are proportional to the model-estimated relative importance of each prey item to a given taxon’s diet. Each prey item is represented by a color (shown by the borders of numbered circles), and the color assigned to an individual snake species is an additive mixture of the colors of the prey items it feeds on. Prey items that commonly co-occur in snake diets are positioned near one another, as are snakes with similar diets. Several rare prey categories (crocodilians, turtles, amphibian eggs, amphibian larvae, and caecilians) are not included here but do not qualitatively alter the appearance of the overall trophic network. The data underlying this figure may be found in doi: 10.5281/zenodo.4446064.

Our analyses reveal striking among-clade variation in rates of diet evolution (Fig 2A), and the inferred trophic network structure shows substantial variation in connectivity among different categories of prey (Fig 2B). Nearly all prey groups have an associated set of specialist predators, but more generalized predators occur almost exclusively among snakes that feed on terrestrial vertebrates. The relative absence of generalized diets that include invertebrates and fishes may stem from the unique adaptations required to subdue and consume these prey and the constraints imposed by small body size and specific macrohabitat associations [51]. Even among more generalized species, however, sampled diets rarely include more than 2 or 3 distinct kinds of prey, and there are clear tendencies for some combinations of prey items to co-occur more commonly in sampled diets than others, reinforcing prior concepts of snake feeding guilds [52–54]. Proposed mechanisms for how these associations arise include the correlated co-occurrence of prey items in the environment as well as chemical and functional similarity of exploited prey [38,55–57].

The inferred trophic structure suggests that vertebrate prey used by snakes can be loosely arranged along a primary axis with terrestrial endotherms (birds and mammals) on one end and aquatic ectotherms (fishes) on the other (Fig 2). Along this axis, terrestrial ectotherms (mainly frogs and squamates) occupy intermediate positions, with amphibians closer to fishes and squamates closer to birds and mammals. At the broadest scale, these associations are likely to be driven, in part, by effects of body size and macrohabitat. Aquatic snakes that prey on fishes regularly encounter frogs that rely on water for reproduction and larval development, for example, and only larger snakes can safely subdue and consume birds and mammals. Many of the commonly recorded invertebrate prey items in sampled snake diets are themselves dangerous predators (centipedes, spiders, and scorpions) that are important sources of mortality in squamate reptiles [58,59], requiring large body size and venom to subdue [60]. Likewise, several groups that are disproportionately used by snakes are heavily defended by shells (snails) and defensive mucosal production (slugs and annelids) and require specialized behaviors, dentition, and oral gland secretions to surmount [61–64]. Interestingly, prior studies indicate that these invertebrate prey groups appear rarely in sampled lizard diets or form minor components of diets rich in other invertebrate groups, contrasting with the extreme level of specialization observed in snakes [65,66].

**Fig 3:**
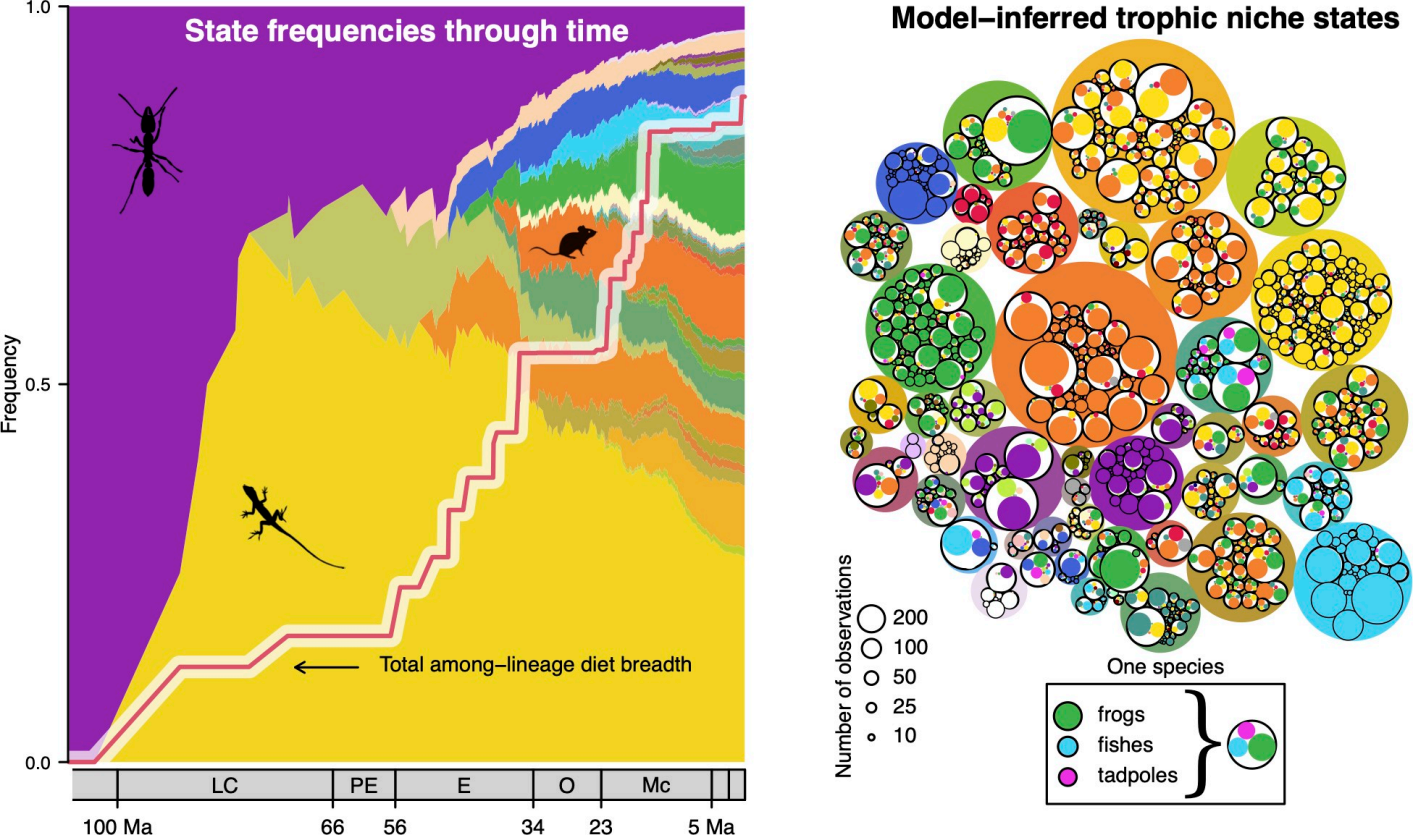
Expansion of trophic diversity during the global radiation of extant snakes. **Left:** Diet-through-time profile showing proportional representation of diet states among lineages at consecutive time slices from the root to the present, with each color representing a distinct multivariate trophic resource state. Colors for each diet state are a proportional mixture of the colors assigned to each prey group under the inferred multinomial distribution (Fig 2). The dominant prey group for a few select diet states is illustrated by the inset silhouettes (e.g., yellow, lizards dominant; purple, insects dominant). **Right:** The full set of diet states and species assigned to each state, showing interspecific variation in sampled prey items assigned to a particular diet state (proportional utilizations of specific prey classes is outlined in Fig 1). Prey groups are colored following the same scheme used in Fig 2. The diversity of snake dietary niches expanded markedly during the Eocene, when reconstructed cladogenetic events mark the origin of many higher taxonomic snake lineages. Figure illustrates the sample from the posterior with the highest probability for *K* = 1,000; additional samples are shown in the Supporting information section (S2, S3, S5, and S6 Figs). There is considerable uncertainty in the precise sequence of overall trophic expansion across snakes, but the qualitative pattern showing an early shift to a specialized vertebrate diet followed by an Eocene expansion in among-lineage diet breadth remains largely unchanged (S6 Fig). Each diet state (left) is plotted such that its age of origin corresponds to the crown clade age of the reconstructed ancestor where it first appears. Inset images from PhyloPic are available under public domain. The data underlying this figure may be found in doi: 10.5281/zenodo.4446064.

Our analysis reconstructs the most recent common ancestor of living snakes as feeding exclusively on invertebrates (mainly insects) with high probability, followed by an early shift to a vertebrate diet (Figs 2 and 3). An insect-feeding ancestor is consistent with phylogenetic relationships among snakes as currently understood: The earliest diverging snake lineages, scolecophidians (blind snakes), comprise a paraphyletic assemblage of species that feed almost exclusively on eusocial insects. How morphologically and ecologically representative blind snakes are of early ancestral snakes is contentious. Some have considered them phenotypically similar to the earliest snakes [67,68], but others have argued that they are highly derived and cannot be considered morphological or ecological analogs of snake ancestors [69,70]. The situation is further obscured by conflicting placements of blind snakes with respect to fossil snakes and other crown group snakes across different datasets and analysis techniques [71]. Regardless, it is clear that the shift to vertebrate feeding happened early in snake evolution, maybe even facilitated by the consequent increase in gut volume resulting from adaptive morphological changes in response to fossorial habits [72].

Modifications in skull morphology associated with the origin of large-gaped snakes (Macrostomata) led to the elaboration of vertebrate feeding strategies [28], and snakes subsequently diversified into many distinct feeding modalities after the K-Pg boundary during the Eocene, a time when squamate communities were beginning to recover from end-Cretaceous extinctions [24] (Fig 3). The tempo of trophic expansion during this time is substantially more rapid than the pattern expected under a null model of ecophenotypic diversification (*P* < 0.05; S2 Fig). Per-lineage dietary niche breadths remained narrow and relatively constant over the same time period (S3 Fig), indicating that the rapid expansion in diet ecospace occupied by snakes was due to repeated transformational shifts in prey preferences and suggesting a possible role for ecological opportunity due to reduced competition in the wake of the K-Pg extinction event.

Our finding that ancestral snake diets were narrowly specialized rejects the idea that many specialized feeding modalities originated from more generalized ancestors (S3 Fig). A pattern of generalists giving rise to specialists was a widely held expectation in early conceptualizations of adaptive radiation [73], but our results suggest that specialists are no less evolutionarily versatile than generalists [74]. This is not to say that no dietary shifts toward highly specialized feeding modalities were preceded by generalized ancestors. For example, in our reconstructions, many egg-eating snakes arose from ancestors inferred to occasionally eat eggs as part of a broader diet, consistent with previous findings [56]. However, in other cases, such stepping-stone–like patterns appear unlikely. For example, 13 of the 15 recorded prey items for the Neotropical dipsadine *Rhachidelus brazili* are bird eggs, but no bird eggs are recorded in 139 prey items from the diets of its 5 closest relatives, which consist largely of lizards, snakes, and mammals. These results suggest the potential for occasional dramatic (rapid) dietary shifts, an inference that is supported by some observations from present-day snake populations. At least one population of Galapagos snakes (*Pseudalsophis*), for instance, has taken to intertidal foraging on coastal fishes [75], a behavior unknown from any other populations or close relatives [76] that lends support to the claim that niche shifts are frequently initiated by changes in behavior [77]. These results are consistent with adaptive landscape models—which predict that “peak shifts” toward new phenotypic optima entail explosive change away from current optima [78]—and suggest that ecological trait divergence is, in some cases, consistent with theoretical expectations developed for morphological data.

**Fig 4:**
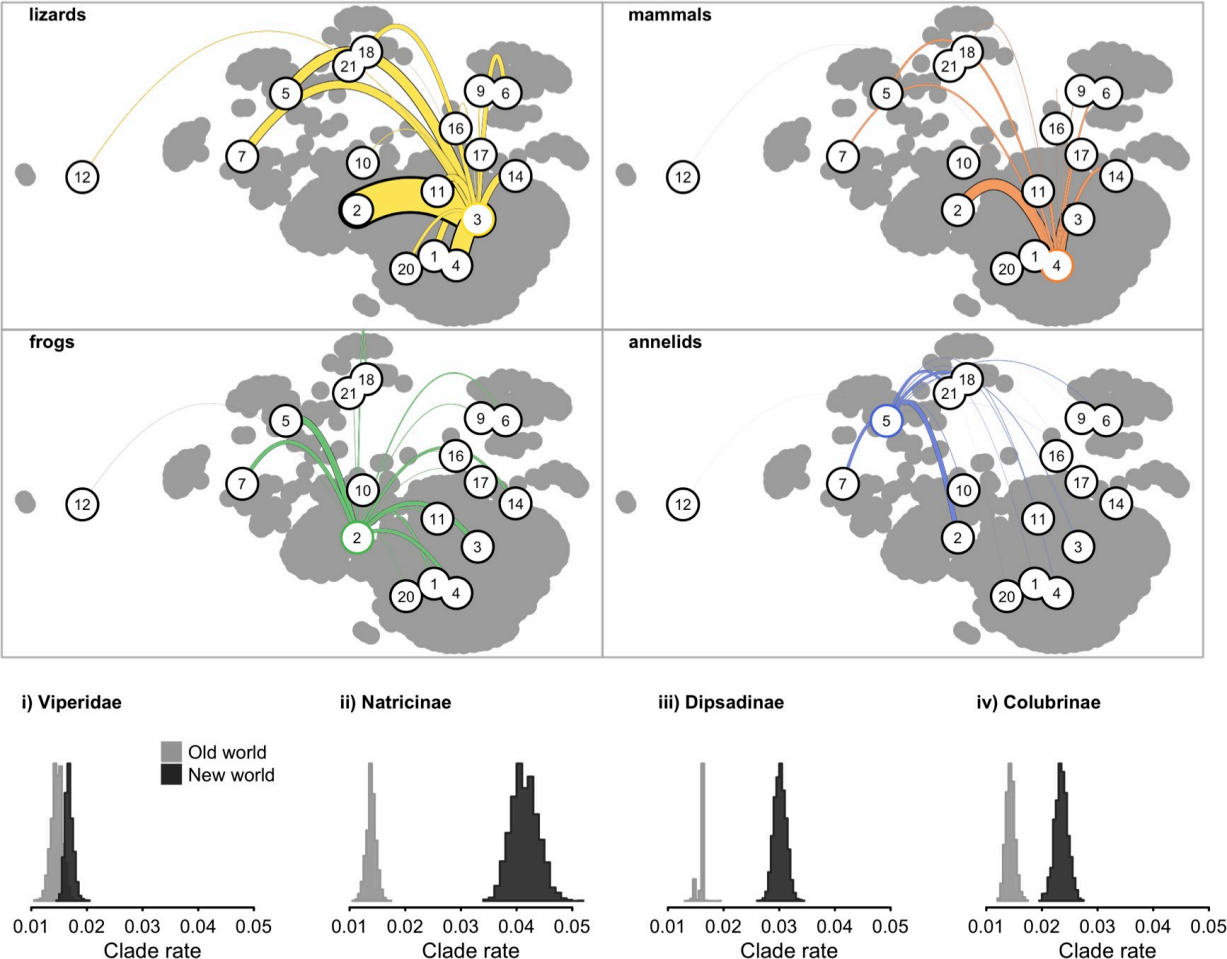
**Evolutionary flux between major trophic resources (top) and rate of trophic evolution (bottom) during the ecological radiation of snakes. Top:** Each subpanel illustrates the average number of transitions between a given resource category (e.g., lizards: upper left, yellow) and all resources (numbered circles; see Fig 2); line thickness is proportional to the number of transitions. Reconstructions of ancestral snake diets were inferred using a Dirichlet-multinomial Markov model, and gains and losses between ancestors and descendants were computed under an optimal transport model (see text for details). Colors and numbers follow the same scheme used in Fig 2. Colored lines depict inferred evolutionary gains of different prey categories from the ancestral prey category highlighted in color, and line widths are proportional to the total number of inferred gains. Transitions from only 4 ancestral prey categories are shown. Numerous independent origins of similar feeding strategies occur across the snake tree of life, often from a lizard-eating ancestor. Gains and losses are unequally distributed among prey categories, and some feeding strategies show much greater turnover than others, suggesting that feeding strategies differ in evolutionary accessibility and versatility. **Bottom:** Reconstructed rates of trophic evolution across 4 major snake radiations indicate that neartic and neotropical (NW) clades exhibit greater net rates of diet evolution than their OW relatives, suggesting that colonization of new biogeographic theaters has been an important source of ecological opportunity in the adaptive radiation of snake diets. In panel (iii), OW relatives include *Stichophanes* (Dipsadinae) and *Pseudoxenodon* (Pseudoxenodontinae). Histograms depict the posterior distribution of average clade rates and are derived from evolutionary flux between different trophic resources (see Methods). The data underlying this figure may be found in doi: 10.5281/zenodo.4446064. NW, New World; OW, Old World.

Our results imply that a striking diversity of trophic modalities are inferred to have originated from a lizard-eating ancestor in a relatively brief period of time (Fig 4, S4 Fig). The evolutionary dynamics of prey switching are quantified using evolutionary flux, a metric that measures gains and losses of different prey groups while accounting for the continuous nature of the dietary niche (Methods). Lizards are abundant in the same terrestrial environments as snakes, and their generally small body size compared with most snakes makes them suitable prey for a broad range of snake body sizes and gape widths. Indeed, many snakes that feed on birds and mammals as adults have juvenile diets comprised of lizards [79], and lizards may have been the target of early selection during the shift to a vertebrate diet. However, there remains considerable uncertainty in the precise sequence of overall trophic expansion across snakes (S5 and S6 Figs).

Our analysis recovers numerous independent origins of similar feeding strategies across the global snake radiation. Notably, independent origins of specialized mammal eaters first appear unambiguously in ancestral states with the most recent common ancestors of vipers, boas, and pythons during the Eocene, a time when rodents (the predominant mammals recorded in snake diets) were spreading and diversifying around the world [80] and consistent with prior suggestions that the rise of mammals, particularly rodents, provided ecological opportunity for the diversification of some snake clades [30,37,81,82]. Perhaps, most remarkably, vermivory (earthworm feeding) has arisen independently in nearly all major snake lineages, including typhlopids (*Acutotyphlops subocularis* [83]), uropeltids, xenodermids (*Achalinus* [84]), pareids (*Xylophis* [85]), viperids (*Atheris barbouri* [86]), homalopsids (*Brachyorrhos* [87]), elapids (*Toxicocalamus* [88] and *Ogmodon* [89]), lamprophiids (*Oxyrhabdium* [90]), natricids, pseudoxenodontids (*Plagiopholis* [91]), dipsadids, and colubrids. Phylogenetic autocorrelation in the proportion of annelids in sampled snake diets is the lowest of all prey categories: Despite a similar number of reconstructed gains, annelids in sampled snake diets show considerably lower phylogenetic clustering than vertebrate prey items like mammals and fishes (S7 Fig). Such differences hint at the possibility that feeding strategies differ in evolutionary accessibility and versatility, and earthworm feeding may be among the most evolutionary and ecologically accessible feeding strategies available to snakes. Alternatively, low phylogenetic autocorrelation may also suggest that vermivory is a so-called “self-destructive” trait and that there are limited opportunities for species diversification for lineages that switch to an earthworm diet [92].

Reconstructed ancestor-descendant diet sequences reveal evidence of elevated rates of change among colubroid snakes, a cosmopolitan clade comprising most of living snake diversity (Fig 2). Rates of change measure the tempo of prey switching by snake lineages and are calculated by dividing the total evolutionary flux among prey groups by the span of time over which it occurred (Methods). A number of key innovations are hypothesized to have facilitated the spectacular diversification of colubroids and their wide range of dietary adaptations, including the decoupling of locomotory and feeding behaviors and the freeing of the mandible from its role in intraoral prey transport [28,30,32]. Within colubroids, some of the fastest rates of dietary change are associated with colonization of the Nearctic and Neotropical regions by the colubrid subfamilies Natricinae, Dipsadinae, and Colubrinae, consistent with observations from other snake clades that show that new biogeographic opportunities spur evolutionary innovation (S8 Fig) [93]. Within natricines, for example, colonization of the New World resulted in a roughly 200% increase in the rate of trophic niche evolution (posterior mean rate) relative to putatively ancestral background rates for the clade (Fig 4, S8 Fig). Similar increases were observed for dipsadines (90%), colubrines (64%), and viperids (15%).

Colubrids show systematically higher net rates of change than non-colubrids (S9 Fig), suggesting that clade-level differences in dietary lability rather than timescaling effects [94–97] play a role in driving trophic rate variation and hinting at the possibility of a general coupling between rates of lineage diversification and rates of trophic evolution. In spite of generally higher rates of trophic innovation in colubrids, however, nearly all feeding modalities observed in the current dataset also occur in other colubroid lineages that diverged prior to the origin of colubrids, indicating that the colubrid mega-radiation has been facilitated more by an ability to exploit existing ecological opportunities rather than by invasion of previously inaccessible trophic niches.

## Conclusions

We demonstrated how primary natural history observations can be integrated with stochastic model–based comparative methods to describe the evolution of complex ecological phenotypes. Although our focus was the ecological diversification of snakes, the methodological and visualization framework we describe can be applied to many multivariate ecological or behavioral data without requiring researchers to first define and circumscribe ecological states among their study taxa. Prior comparative analyses have emphasized the deep historical roots of dietary differences observed in lizard [65] and snake [98] communities. Our results extend this perspective by explicitly quantifying reconstructed evolutionary dynamics of historical ecological transitions, revealing a dramatic ecological expansion in occupied diet space beginning in the mid-Eocene and elevated rates of trophic innovation following colonization of the Nearctic and Neotropical realms by major snake lineages.

By using fundamental observations of organisms in nature to make quantitative inferences about the macroevolution of a complex ecological trait, our study suggests new ways of integrating natural history data into comparative biology. A renewed emphasis on causal modeling in macroevolution [99] together with a concerted effort to collect and analyze primary natural history observations at broad phylogenetic scales promises to open the door to new datasets and novel lines of inquiry into the macroevolution of complex phenotypes like diet, habitat, movement patterns, and demography. Because primary natural history observations capture significantly more of the complexity in natural communities than categorical descriptors, their integration with phylogenetic comparative methods may hold the key to long-standing questions about the evolution of specialization, how patterns of resource use affect the evolution of phenotypes, or how and why species evolve new ways of life.

The data included in the present study represent the combined effort and observations of numerous field workers over many decades. Even with thousands of prey observations, however, there is still a substantial fraction of snake diversity that is poorly known ecologically. The 882 species included in our study cover less than a quarter of living snake diversity, and fully half the species in the dataset are represented by only 12 or fewer observations. Our results call attention to this deficit and highlight a critical need to gather more natural history data to help advance our understanding of how complex ecological traits evolve.

## Methods

### Data acquisition and prey categorization

We compiled a dataset of prey items observed in sampled snake diets through an extensive review of the primary literature and organized observations into a publicly available database that is accessible through the R package SquamataBase [100]. For the present analysis, we categorized observations into 22 different groups according to higher level prey taxonomy. These prey groups (labels: Fig 2) are commonly used in the published natural history literature, and the presence of unambiguous dietary specialists for most prey groups indicates that they capture relevant variation in snake prey preferences. The dataset uses a composite picture of the prey spectrum sampled by individual species by aggregating records across different sources of intraspecific variation (e.g., age, sex, geography, and activity season). This decision was motivated by the broad phylogenetic scope of the current study and uneven sampling across species, but there remain important opportunities for incorporating different sources of intraspecific variation into comparative analyses of this sort. For comparative analysis, we used the phylogenetic hypothesis from [47]. The maximum likelihood topology was inferred using mitochondrial and nuclear sequence data from 5,415 squamate species, of which 1,583 were snakes, and time calibration was performed under a relaxed clock model with fossil node age constraints. Full details are given in [47].

### Probabilistic reconstruction of diet states and trophic niche evolution

To reconstruct the history of snake trophic niche evolution, we used a new comparative method that we previously developed for modeling the evolution of complex ecological phenotypes. A theoretical description and simulation-based validation of the method are given in [46]. Briefly, the model assumes that the diets for each species follow a multinomial probability distribution over a set of prey categories. Individual prey records are treated as samples from these “true” diet states. The diet state for a species is therefore hidden, and estimates of dietary niche states for species are subject to sampling variation. Each internal node in a phylogeny is also described by an unobserved diet state, and changes between diet states along the branches of a phylogeny are modeled as a Markov process where distinct multinomial distributions correspond to different “character” states (Fig 1).

More formally, each extant species in a phylogeny can be characterized by a vector ***x*** that describes its proportional utilization of the 22 different diet categories. If we denote the full set of such vectors by ***X***, the goal of the method is to both estimate ***X*** and to extend ***X*** so that hypothetical ancestral species, represented by internal nodes of the phylogeny, are also assigned proportional utilization vectors. We denote the full set of vectors for terminal and internal nodes by $\hat{X}$. By assumption, the proportional utilization vectors reconstructed for ancestors must be represented among the set observed in ***X***. This is enforced by assuming that ***X*** contains at most *K* unique proportional utilization vectors corresponding to distinct trophic niche states (the actual number discovered by the method may be less than *K*). Because the observed data consist only of counts, the method samples a range of ***X*** that confer high probability on the observed count data under a Dirichlet-multinomial sampling model with hyperparameter α, and the method probabilistically reconstructs all ancestral vectors that are consistent with ***X***. As a result, the method returns a posterior distribution for $\hat{X}$ that accommodates uncertainty in both observed and ancestral trophic niche states. We used an uninformative Dirichlet prior (α = 1) and set *K* = 1,000, a relatively high value that favors more parsimonious evolutionary histories due to the stronger penalty incurred with each event of character state change. Gibbs sampling was performed for 30,000 iterations, and every 10th sample was recorded for a total of 3,000 posterior samples. Posterior averages reported in the main text were computed after discarding the first 500 samples. Likelihood and parameter traces are provided in the Supporting information section (S10 Fig). To examine the influence of *K*, we ran a second set of analyses after the first, but setting *K* = 50 as this was the modal number of character states represented among the terminal nodes when *K* was set equal to 1,000. All analyses were performed in the R package macroevolution [46].

### Estimating evolutionary fluxes and net rates of change

To quantify the dynamics of evolutionary changes in snake trophic niches, we estimated average evolutionary flux between diet categories for each branch in the phylogeny under an optimal transport model. In this context, evolutionary flux between 2 diet categories corresponds to a fractional number of gains or losses that must occur to transform an ancestral diet state into a descendant diet state. Specifically, for a given ancestor (*u*) and immediate descendant (*v*), we computed the matrix $U_{\hat{x}(u),\hat{x}(v)}$ that transformed an ancestral diet $\hat{x}(u)$ into a derived diet $\hat{x}(v)$ (where $\hat{x}(u)\neq \hat{x}(v)$) at a minimum total cost $d_{U}(\hat{x}(u),\hat{x}(v))$. Note that the rows of $U_{\hat{x}(u),\hat{x}(v)}$ must sum to $\hat{x}(u)$ and the columns to $\hat{x}(v)$. The optimal transformation cost is defined as

$$d_{U}(\hat{x}(u),\hat{x}(v))=\underset{U_{\hat{x}(u),\hat{x}(v)}}{\min}\sum_{jk}U_{jk}C_{jk},$$

where the sum runs over all pairs of diet categories. The elements of $U_{\hat{x}(u),\hat{x}(v)}$ describe how much proportional utilization of each diet category in an ancestor must be transformed into proportional utilization of each diet category in a descendant. The matrix ***C*** assigns a value to the cost of transforming a unit of one diet category into a unit of another and must be directly specified. The matrix ***C*** was considered fixed, with diagonal elements set to 0 and off-diagonal elements set to 1. With this specification, the elements of $U_{\hat{x}(u),\hat{x}(v)}$ can be thought of as the effective number of gains and losses that take place during an evolutionary transition from one diet to another. In the extreme case, when ancestor and descendant are pure specialists on different prey groups, only a single element of $U_{\hat{x}(u),\hat{x}(v)}$ is nonzero and that element will equal 1. Matrices $U_{\hat{x}(u),\hat{x}(v)}$ were computed for each branch using the Sinkhorn–Knopp algorithm [101]. To accommodate uncertainty in ancestral diet states, we computed a weighted version of ***U*** as

$$U_{w}=\frac{\sum_{qr}w_{qr}U_{\hat{x}(u)=q,\hat{x}(v)=r}}{\sum_{qr}w_{qr}},$$

where the sum runs over all pairs of diet states and where

$$\begin{array}{c} w_{qr}=P(\hat{x}(v)=r,\hat{x}(u)=q|D)\\ =P(\hat{x}(v)=r|\hat{x}(u)=q,D)P(\hat{x}(u)=q|D). \end{array}$$

Here, ***D*** represents the diet observations recorded for terminal taxa in the phylogeny. For $P(\hat{x}(u)=q|D)$, we used the marginal ancestral state probability that node *u* had diet state *q*, and we computed $P(\hat{x}(v)=r|\hat{x}(u)=q,D)$ using the stochastic mapping algorithm [102]. We then averaged ***U***_*w*_ over all posterior samples to assign each branch a single flux matrix.

We estimated the net rate of trophic niche evolution for each clade by calculating the average total flux among prey categories within a clade divided by the total branch length of all lineages in the clade. The average total evolutionary flux among prey categories over a single branch is simply ∑_*jk*_*U*_*jk*_(1−*I*_*jk*_), where *U*_*jk*_ is an element of the branch-specific ***U***_*w*_ matrix, *I*_*jk*_ is an indicator variable that equals 1 when *j = k*, and the sum runs over all pairs of prey categories. We calculated the total flux for a clade by summing the total branch-specific flux over all branches descended from the clade’s root. We used these clade rates to assign each terminal node a tip rate, which we computed as the weighted average of all clade rates on the phylogenetic path leading from the tip to the root. Tip rates calculated with this method are shown in Fig 3. The weights attached to each clade rate increase as they approach the root, which relaxes each tip rate back to the overall average clade rate computed from the entire phylogeny. The rationale for assigning higher weights to more ancient clades (as opposed to the DR statistic [103], which weights younger lineages more heavily) is that it acts as a form of regularization and reduces the volatility in tip rates that results from evolutionary events occurring on shorter branches near the present. The strength of regularization depends on how quickly weights increase toward the root, but the overall qualitative picture of variation in tip rates is unchanged by different weighting schemes (S11 Fig). Computing tip rates in this fashion helps relax the assumption of rate homogeneity imposed by the underlying model, and similar tip rates have been shown to effectively capture heterogeneous speciation rate dynamics in other contexts [104].

### Visualizing diet states and trophic niche evolution

To visualize multivariate diet states, we assigned each prey group a color (Fig 2). Colors for each diet state were then a proportional mixture of the colors assigned to each prey group under the inferred multinomial distribution. Specifically, the component color value (red, green, or blue) for a diet state was set equal to ∑_*j*_*p*_*j*_*c*_*j*_, where the sum runs over all prey categories. Here, *p*_*j*_ is the estimated proportion of prey item *j* and *c*_*j*_ is the component color value assigned to the prey item. To visualize the history of trophic changes during snake evolution, branches of the phylogeny were painted with these colors. To accommodate uncertainty within a posterior sample, colors for internal nodes were averaged over different diet states using marginal ancestral state probabilities. Colors were then averaged over all posterior samples for a final set of colors (Fig 2).

To visualize the overall trajectory of trophic expansion, we also visualized single posterior samples using stochastic map realizations to create diet-through-time profiles (Fig 4, S5 and S6 Figs). For each internal node *u*, we calculated the proportional representation of different diet states among all nodes as old as or older than *u*. The total among-lineage diet breadth for the time slice defined by internal node *u* was then calculated as ∑_*j*_*D*_*j*_, where *D*_*j*_ is the largest absolute difference in proportional utilization of a single prey group among the set of nodes at least as old as node *u*, and the sum runs over all prey categories.

### Permutation analysis of dietary ecospace expansion

We developed a null model of ecophenotypic diversification to assess how the tempo of dietary ecospace expansion in snakes compares to expectations when ecological opportunity is random with respect to time and phylogeny. For each of the top 500 most probable posterior samples, we first used the marginal ancestral state probabilities to assign each internal node an expected multinomial distribution of proportional prey utilization. We then computed the observed trajectory of among-lineage diet breadth expansion using the approach described above. To compute expected trajectories of among-lineage diet breadth expansion, for each posterior sample, we held the marginal ancestral state probabilities fixed for all nodes but randomized the model-estimated multinomial distributions among states. This preserves phylogenetic signal while allowing ecological opportunity to be random with respect to time and phylogeny. We generated 99 randomizations for each posterior sample and computed trajectories of total among-lineage diet breadth expansion for each permutation using the same approach as for the observed data. For each internal node, we then assessed how strongly the observed among-lineage diet breadth departed from expectations by calculating the proportion of permutations with a total among-lineage diet breadth at least as large as the observed total among-lineage diet breadth.

## Supporting information

S1 Fig Temporal distribution of reconstructed speciation events in the maximum likelihood DNA phylogeny of extant snakes from [47]. Note that these speciation times pertain only to surviving clades. Early snake clades may have diversified without leaving present-day descendants. Regardless, 99% of snake speciation events with survivors in the present-day postdate the end-Cretaceous extinction event.
(PNG)

S2 Fig Dietary ecospace expansion during the global radiation of snakes. The left column shows the accumulation of among-lineage diet breadth through time. The red line is the observed among-lineage diet breadth for all lineages as old as or older than the *x*-coordinate, and the blue envelope depicts the range of curves possible under a null model. The middle column expresses the observed curve as a rate, with values >1 indicating that total diet breadth is accumulating faster than expected relative to the null model. The null model holds the state labels fixed at all nodes but permutes the multinomial distributions among states, so that phylogenetic signal is preserved while ecological opportunity is allowed to be random with respect to time and phylogeny. The right column quantifies the departure of the observed curve from the expectation as a *P*-value, calculated as the proportion of permutations that achieve an among-lineage diet breadth at least as large as the observed. The horizontal dashed line is *P* = 0.05. The diversity of snake dietary niches expanded markedly during the Eocene beginning 60 Mya, when reconstructed cladogenetic events mark the origin of many higher taxonomic snake lineages. Secondary pulses of trophic innovation occur in the Miocene beginning around 20 Mya when the Nearctic and Neotropical realms were colonized by OW ancestors. This is true regardless of whether the prior model includes *K* = 1,000 (top row) or *K* = 50 (bottom row) character states. OW, Old World.
(PNG)

S3 Fig Change in per-lineage diet breadth over time during the global radiation of snakes. The black curve depicts the average diet breadth for all lineages as old as or older than the *x*-coordinate, and the gray envelope bounds the minimum and maximum diet breadths. Average diet breadth has remained relatively narrow through time, but descendant diets show a trend toward greater generalization. This is true regardless of whether the prior model includes *K* = 1,000 (left) or *K* = 50 (right) character states.
(PNG)

S4 Fig Average number of evolutionary gains and losses among different prey categories in snake diets. Reconstructions of ancestral snake diets were inferred using a Dirichlet-multinomial Markov model, and gains and losses between ancestors and descendants were computed under an optimal transport model (see main text for details). Each cell in the matrix depicts the number of times a prey category in a descendant diet originated from a prey category in an ancestral diet. Point sizes are proportional to the total number of gains/losses. Numerous independent origins of similar feeding strategies occur across the snake tree of life, often from a lizard-eating ancestor. Gains and losses are unequally distributed among prey categories, and some feeding strategies show much greater turnover than others, suggesting that feeding strategies differ in evolutionary accessibility and versatility.
(PDF)

S5 Fig Examples of diet-through-time profiles generated from a single stochastic character map realization from each of the 25 most probable posterior samples for *K* = 1,000. Dashed vertical line depicts the K-Pg boundary. See Fig 3 in the main text for additional details.
(PNG)

S6 Fig Examples of diet-through-time profiles generated from a single stochastic character map realization from each of the 25 most probable posterior samples for *K* = 50. Dashed vertical line depicts the K-Pg boundary. There is considerably more uncertainty around the diet states of Mesozoic ancestors compared with *K* = 1,000 (S5 Fig). See Fig 3 in the main text for additional details.
(PNG)

S7 Fig Phylogenetic clustering (Moran’s I) of the relative importance of different prey categories. Boxplot widths for different prey categories are proportional to the estimated average number of evolutionary gains (numbers along the top margin). Annelids show the lowest levels of clustering, a consequence of their widespread phylogenetic distribution. More restricted prey categories have higher levels of clustering (e.g., mammals and insects). These numbers are potentially impacted by sampling effects. For example, some worm-eating clades have many species (e.g., *Atractus* and *Calamaria*) but are under-represented in the dataset. In this case, more complete sampling of these clades would be expected to increase Moran’s I.
(PNG)

S8 Fig Rate contrasts for major snake radiations show that neartic and neotropical (NW) clades exhibit greater net rates of diet evolution than their OW relatives, suggesting that colonization of new biogeographic theaters has been an important source of ecological opportunity in the adaptive radiation of snake diets. In panel (c), OW relatives include *Stichophanes* (Dipsadinae) and *Pseudoxenodon* (Pseudoxenodontinae). Histograms depict the posterior distribution of average clade rates (see Methods) for the highlighted lineages. NW, New World; OW, Old World.
(PDF)

S9 Fig Net rates of diet evolution for all clades in the phylogeny [47] used in the present study show that, in general, colubrids have the highest rates of all snakes, even after controlling for effects of overall lineage length. This pattern arises mainly from the leveraging effects of dipsadines and natricines, which evolved lots of dietary diversity and speciated quickly upon arrival in the Nearctic and Neotropics.
(PNG)

S10 Fig Likelihood (left) and parameter (middle) traces reveal good mixing of the Gibb’s sampler. The number of distinct dietary niches (right) sampled during the run is far less than the number of species (882), indicating that many sampled snake diets are indistinguishable from one another given the level of sampling. The highlighted portions correspond to the samples that were used to form posterior average summaries mentioned in the main text.
(PNG)

S11 Fig Different weighting schemes (left) applied to the clade rates shown in S9 Fig and their effect on tip rates (right). Tip rates are calculated as a weighted average of all clade rates on the phylogenetic path leading back to the root. While the weighting scheme influences the strength of relaxation toward the overall average rate, the qualitative pattern of tip rates is relatively unchanged by choice of weighting scheme. Tip rates in the main text (Fig 2) correspond to scheme *w* = 1.
(PNG)

S12 Fig Maximum a posteriori multinomial distributions underlying the diet states shown in main text Fig 3. In the left column, each row represents the model-inferred prey use distribution underlying a particular diet state. In the right column, each circular cluster depicts the set of species assigned to a model-inferred diet state. Row numbers (left) and numbers inset below each cluster (right) correspond. Dietary states are identical to those shown in Fig 3. Circles outlined in black represent individual snake species, and subcircles within those circles represent different prey use observations, with circle size proportional to the number of observations. For example, state 12 (right panel; second row from top) represents an annelid specialist, with >0.95 of the multinomial distribution (left panel) concentrated on the annelid prey category. A total of 35 species were assigned to this state, visually represented by the outlined subcircles within state 12 (right panel); see main text Fig 1 for further interpretive information. In contrast, state 24 (left panel; sixth row from bottom) is a generalist, with approximately equal probabilities spread across frog, lizard, and mammal prey categories.
(PNG)

S13 Fig As for Fig 2 of the main text but with *K* = 50. Note that the overall pattern is unchanged with the exception of muddier colored branches near the root, which reflects the greater uncertainty surrounding the diets of Mesozoic ancestors when *K* = 50 (cf S5 and S6 Figs).
(PNG)

S14 Fig As for Fig 4 of the main text but with *K* = 50. Note that greater uncertainty for Mesozoic ancestral states when *K* = 50 (cf S5 and S6 Figs) causes some posterior samples to favor early fish-eating ancestors, which increases the frequency of evolutionary transitions away fish diets relative to results in Fig 4 of the main text.
(PNG)
