# Supplementary figures and images for: Rapid increase in snake dietary diversity and complexity following the end-Cretaceous mass extinction
Source: PLoS Biol. 2021 Oct 14;19(10):e3001414. doi: 10.1371/journal.pbio.3001414 (PMC8516226; doi:10.1371/journal.pbio.3001414)

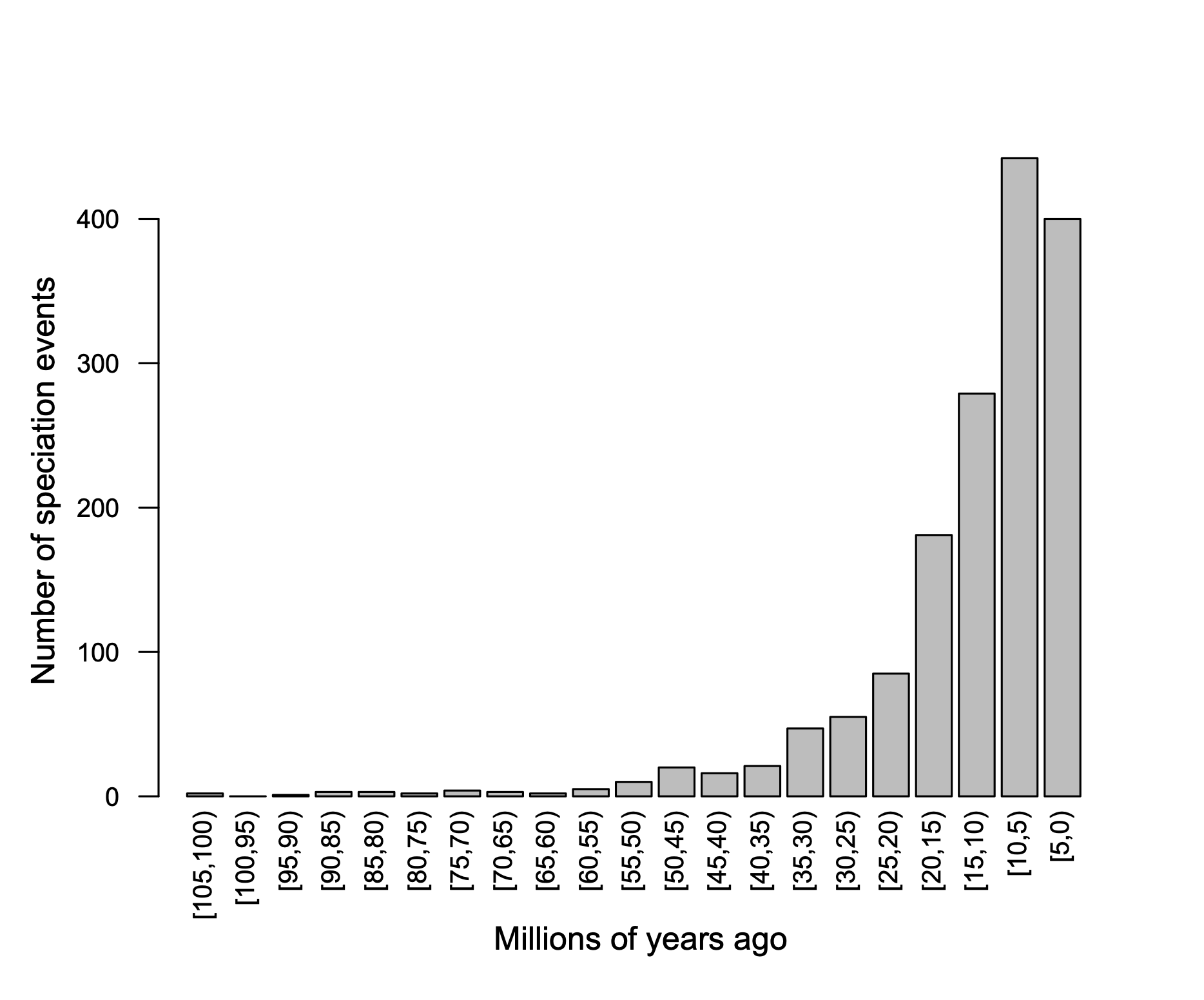

Supplement: S1 Fig — Note that these speciation times pertain only to surviving clades. Early snake clades may have diversified without leaving present-day descendants. Regardless, 99% of snake speciation events with survivors in the present-day postdate the end-Cretaceous extinction event. (PNG) [file pbio.3001414.s001.png]

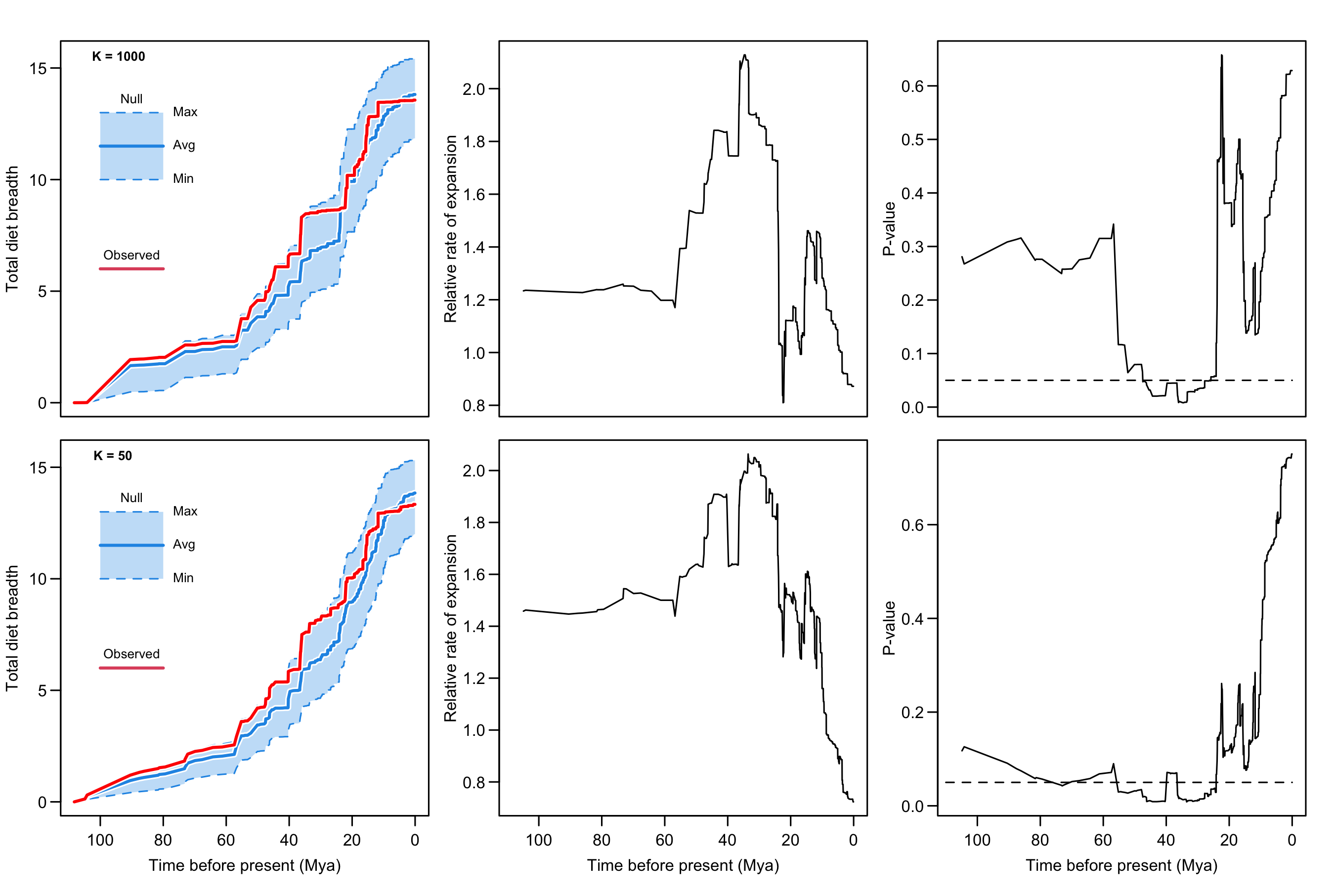

Supplement: S2 Fig — The left column shows the accumulation of among-lineage diet breadth through time. The red line is the observed among-lineage diet breadth for all lineages as old as or older than the x-coordinate, and the blue envelope depicts the range of curves possible under a null model. The middle column expresses the observed curve as a rate, with values >1 indicating that total diet breadth is accumulating faster than expected relative to the null model. The null model holds the state labels fixed at all nodes but permutes the multinomial distributions among states, so that phylogenetic signal is preserved while ecological opportunity is allowed to be random with respect to time and phylogeny. The right column quantifies the departure of the observed curve from the expectation as a P-value, calculated as the proportion of permutations that achieve an among-lineage diet breadth at least as large as the observed. The horizontal dashed line is P = 0.05. The diversity of snake dietary niches expanded markedly during the Eocene beginning 60 Mya, when reconstructed cladogenetic events mark the origin of many higher taxonomic snake lineages. Secondary pulses of trophic innovation occur in the Miocene beginning around 20 Mya when the Nearctic and Neotropical realms were colonized by OW ancestors. This is true regardless of whether the prior model includes K = 1,000 (top row) or K = 50 (bottom row) character states. OW, Old World. (PNG) [file pbio.3001414.s002.png]

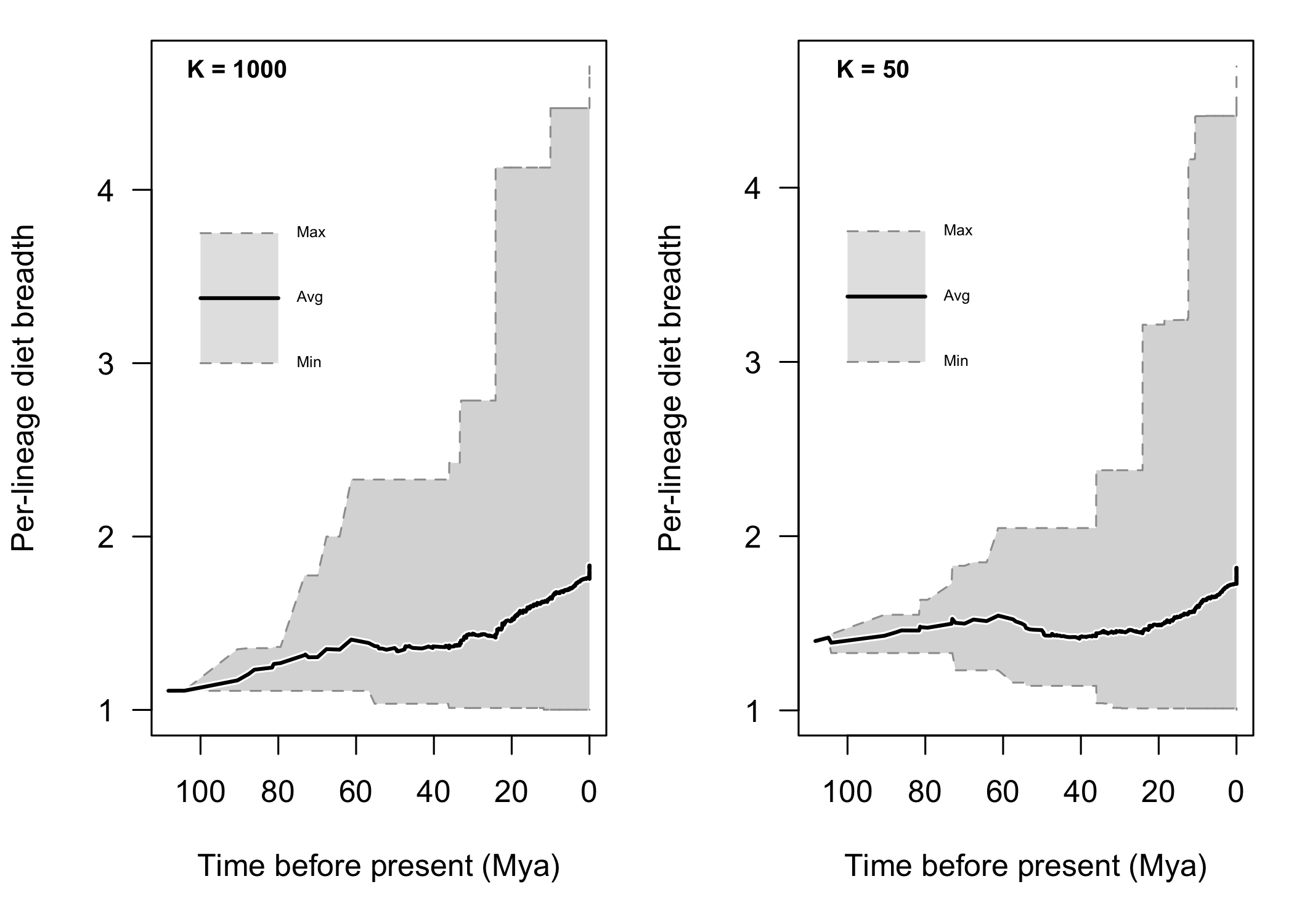

Supplement: S3 Fig — The black curve depicts the average diet breadth for all lineages as old as or older than the x-coordinate, and the gray envelope bounds the minimum and maximum diet breadths. Average diet breadth has remained relatively narrow through time, but descendant diets show a trend toward greater generalization. This is true regardless of whether the prior model includes K = 1,000 (left) or K = 50 (right) character states. (PNG) [file pbio.3001414.s003.png]

Derived (gains)

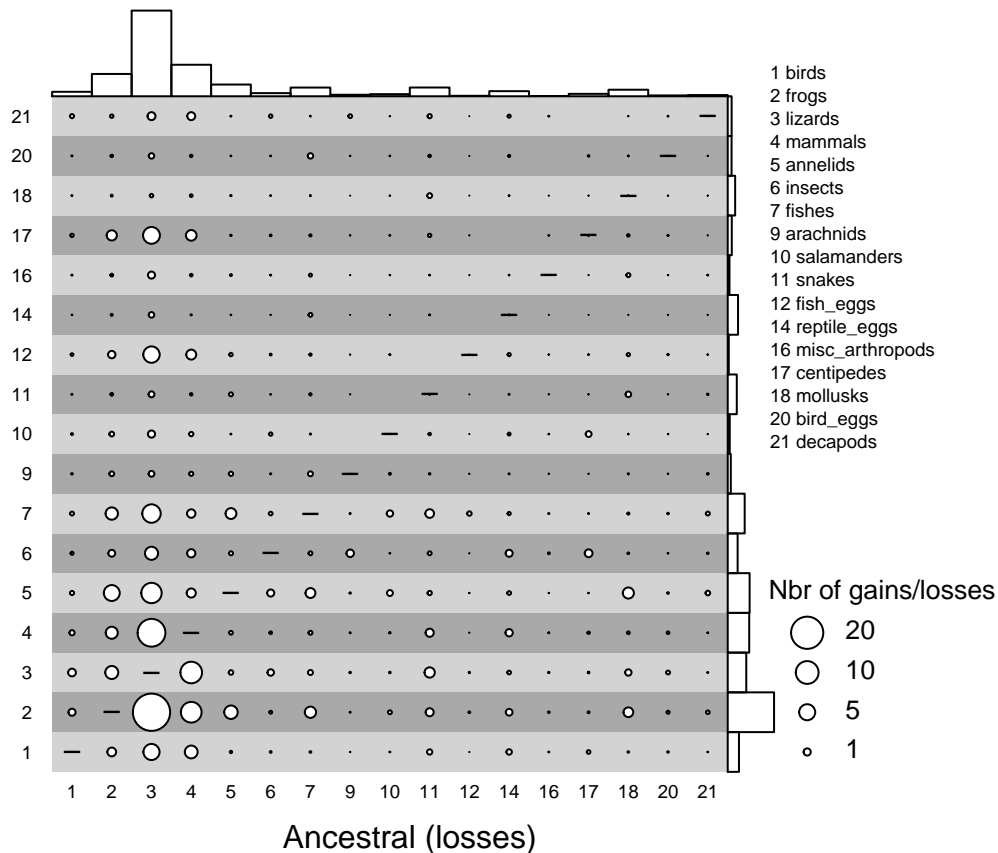

Supplement: S4 Fig — Reconstructions of ancestral snake diets were inferred using a Dirichlet-multinomial Markov model, and gains and losses between ancestors and descendants were computed under an optimal transport model (see main text for details). Each cell in the matrix depicts the number of times a prey category in a descendant diet originated from a prey category in an ancestral diet. Point sizes are proportional to the total number of gains/losses. Numerous independent origins of similar feeding strategies occur across the snake tree of life, often from a lizard-eating ancestor. Gains and losses are unequally distributed among prey categories, and some feeding strategies show much greater turnover than others, suggesting that feeding strategies differ in evolutionary accessibility and versatility. (PDF) [file pbio.3001414.s004.pdf]

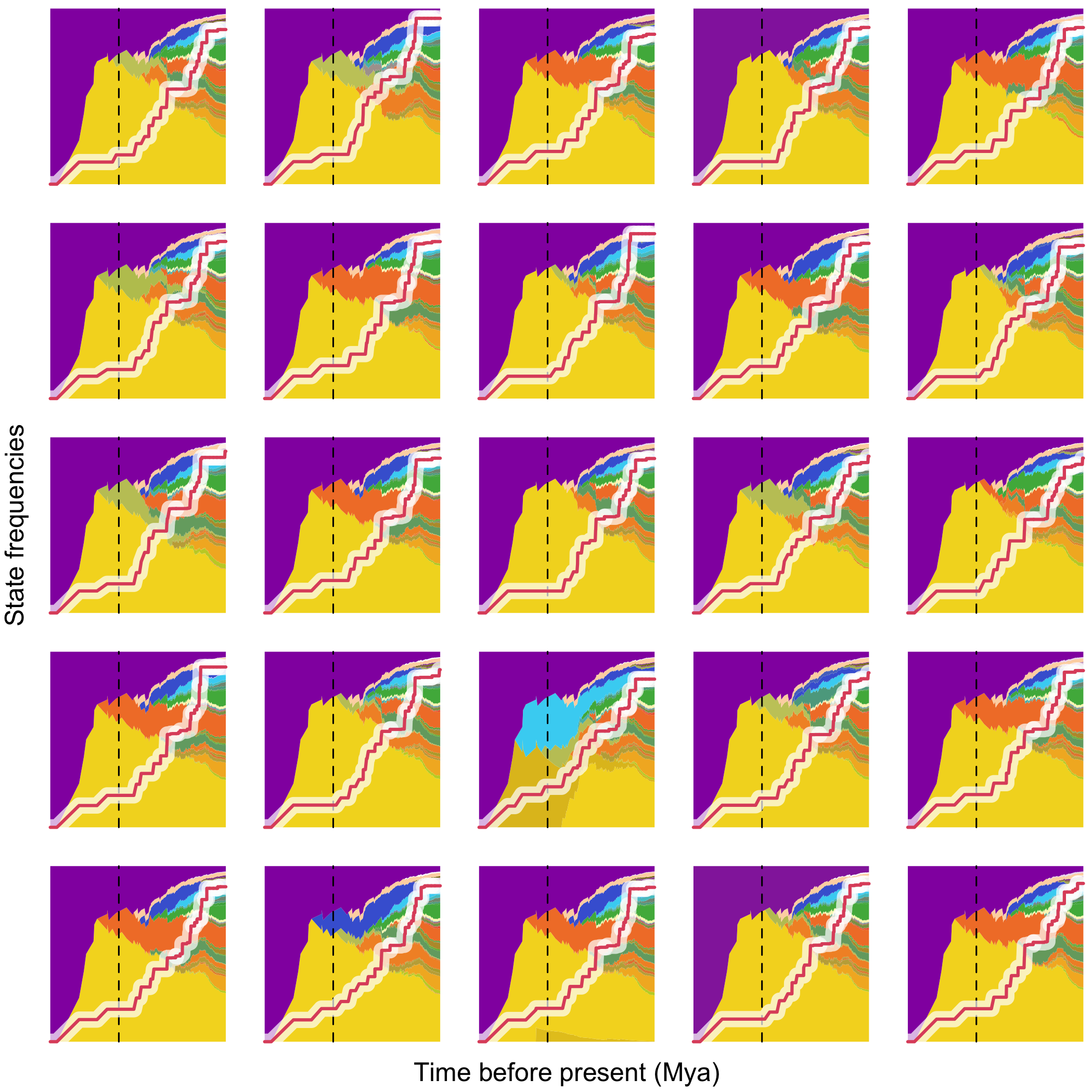

Supplement: S5 Fig — Dashed vertical line depicts the K-Pg boundary. See Fig 3 in the main text for additional details. (PNG) [file pbio.3001414.s005.png]

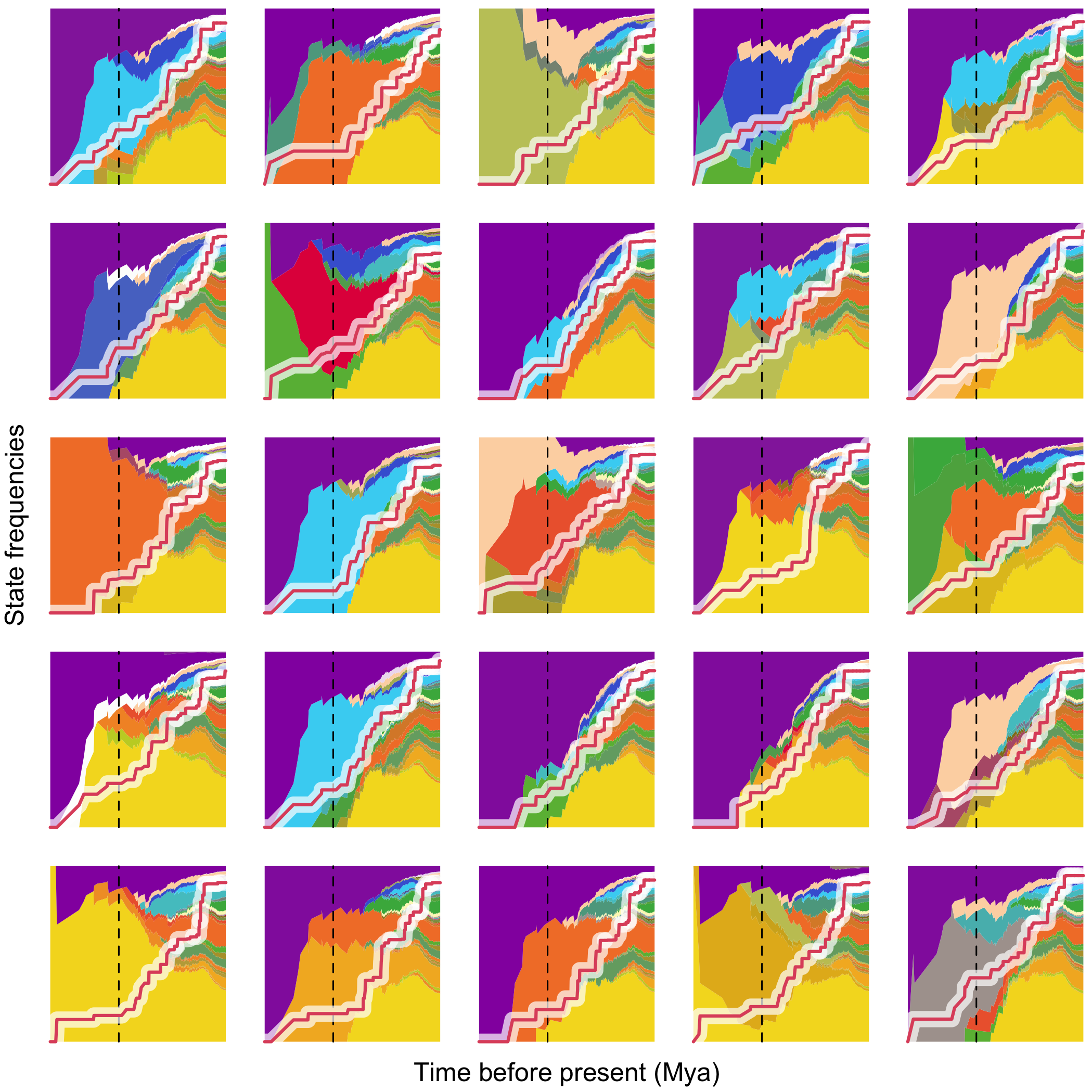

Supplement: S6 Fig — Dashed vertical line depicts the K-Pg boundary. There is considerably more uncertainty around the diet states of Mesozoic ancestors compared with K = 1,000 (S5 Fig). See Fig 3 in the main text for additional details. (PNG) [file pbio.3001414.s006.png]

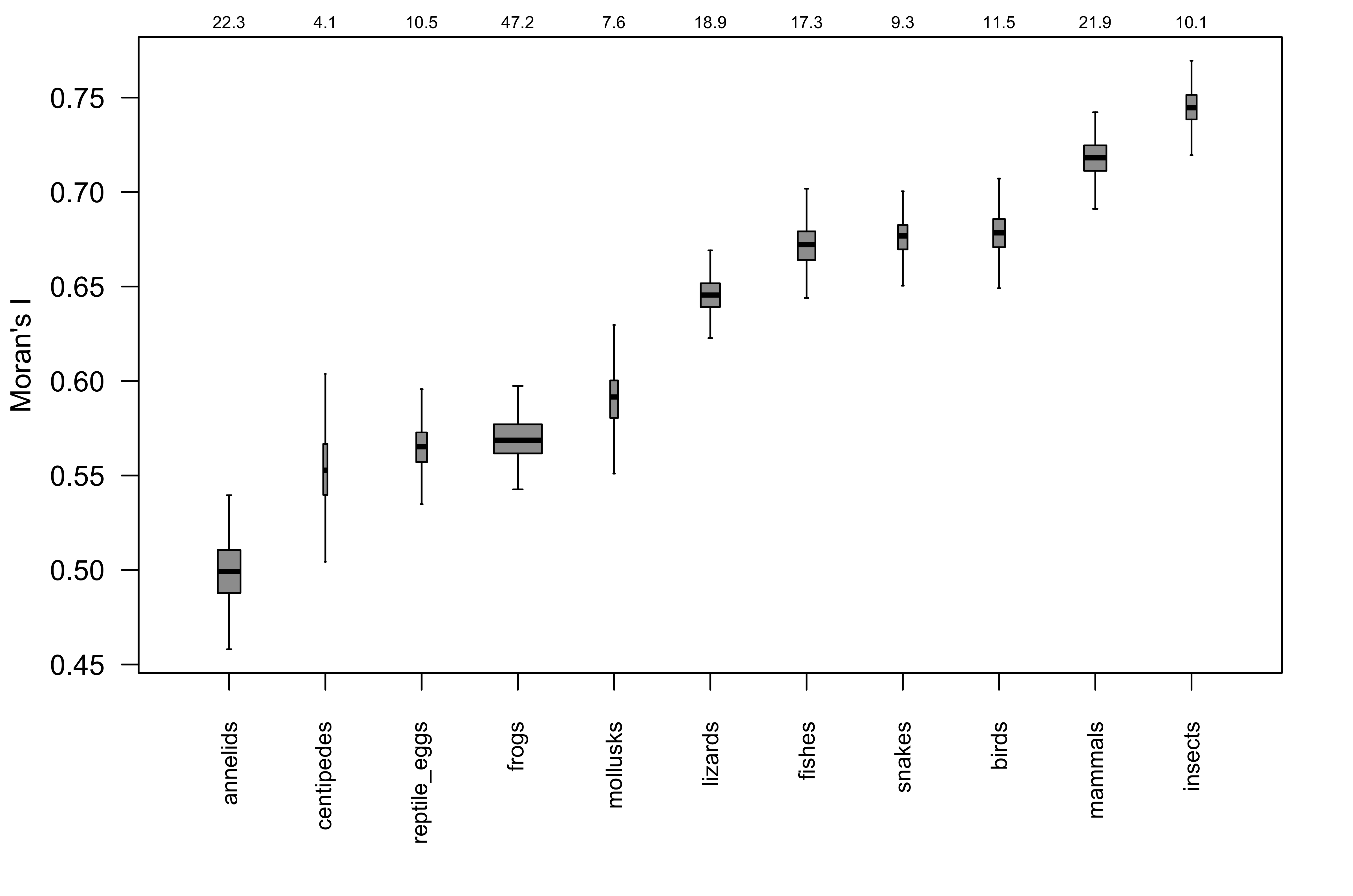

Supplement: S7 Fig — Boxplot widths for different prey categories are proportional to the estimated average number of evolutionary gains (numbers along the top margin). Annelids show the lowest levels of clustering, a consequence of their widespread phylogenetic distribution. More restricted prey categories have higher levels of clustering (e.g., mammals and insects). These numbers are potentially impacted by sampling effects. For example, some worm-eating clades have many species (e.g., Atractus and Calamaria) but are under-represented in the dataset. In this case, more complete sampling of these clades would be expected to increase Moran’s I. (PNG) [file pbio.3001414.s007.png]

a) Viperidae

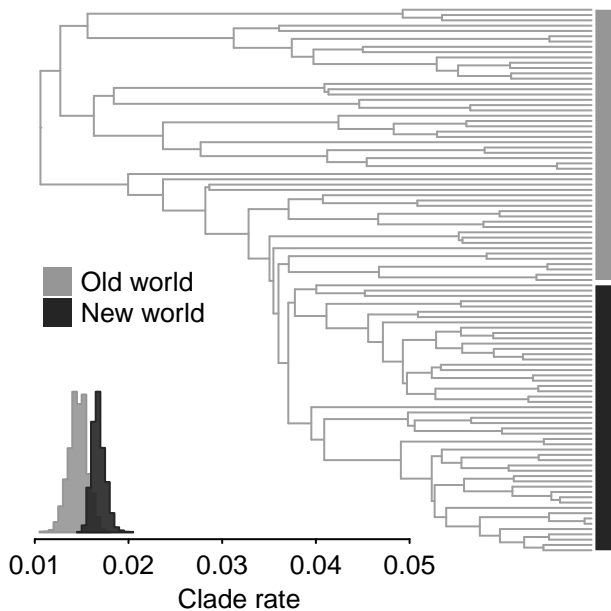

b) Natricinae

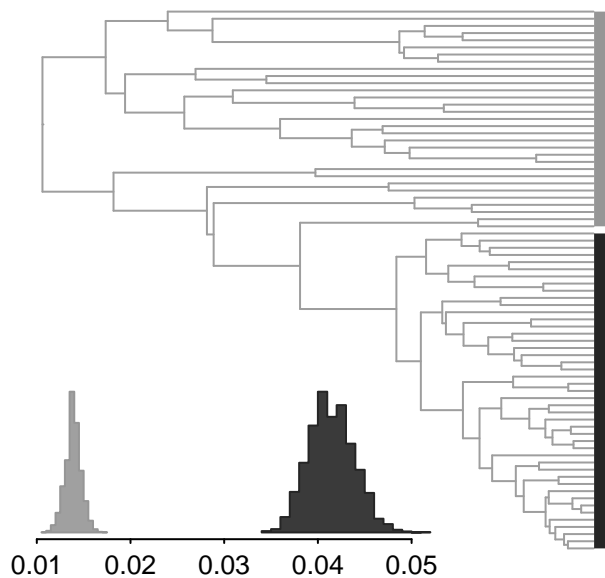

c) Dipsadinae

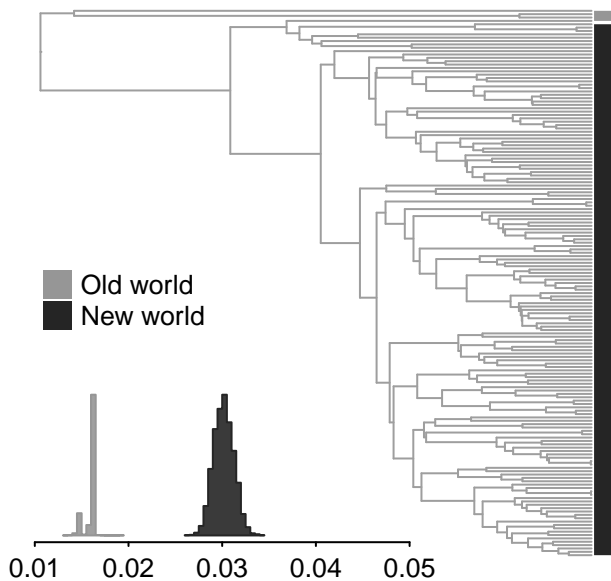

d) Colubrinae

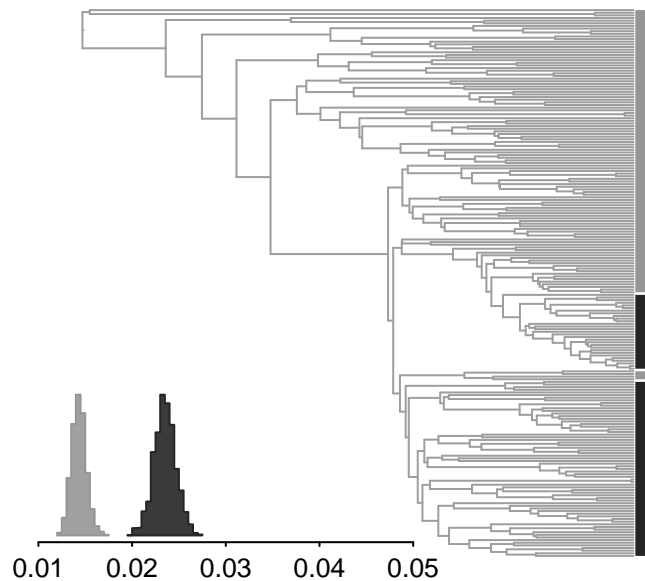

Supplement: S8 Fig — In panel (c), OW relatives include Stichophanes (Dipsadinae) and Pseudoxenodon (Pseudoxenodontinae). Histograms depict the posterior distribution of average clade rates (see Methods) for the highlighted lineages. NW, New World; OW, Old World. (PDF) [file pbio.3001414.s008.pdf]

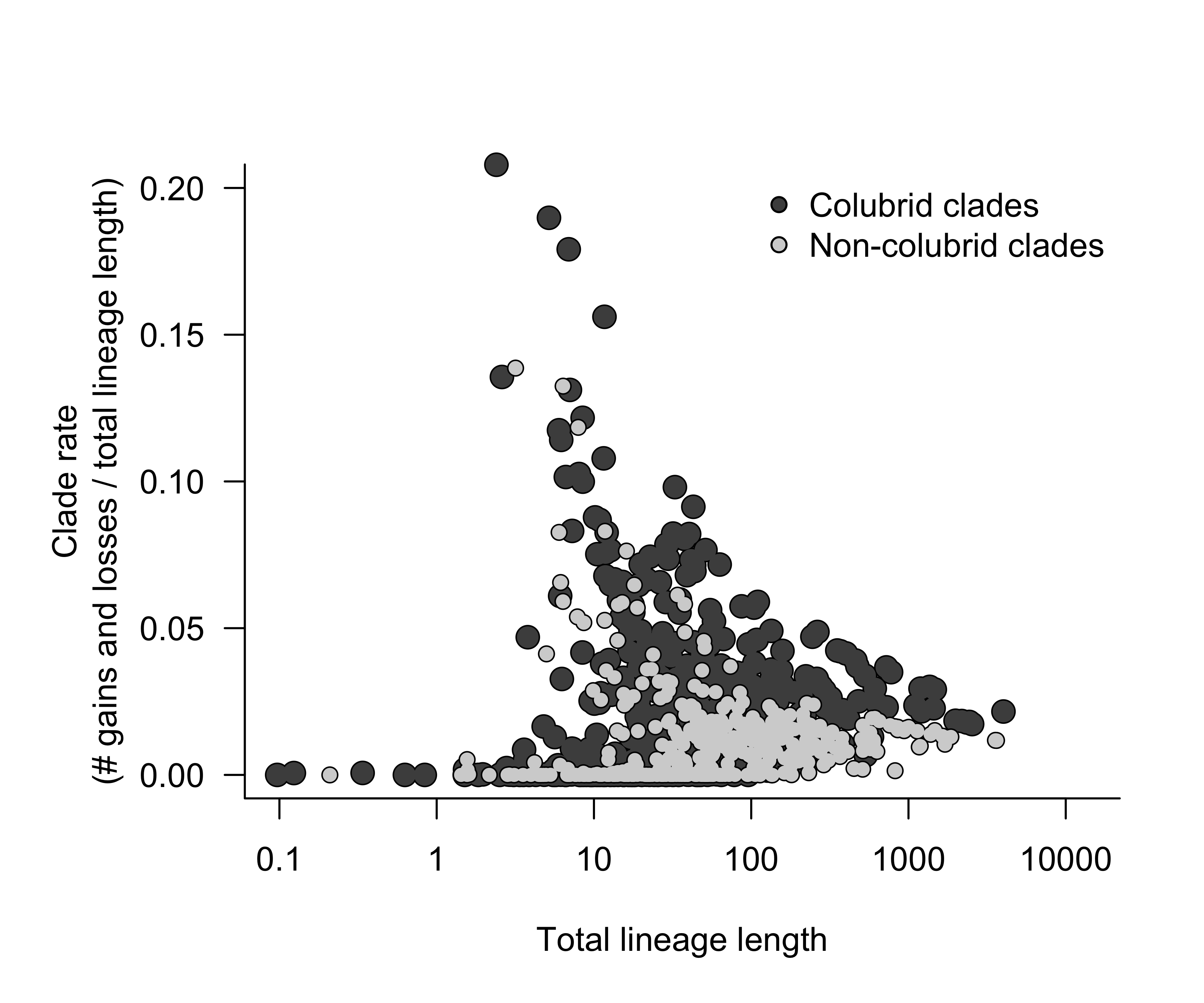

Supplement: S9 Fig — This pattern arises mainly from the leveraging effects of dipsadines and natricines, which evolved lots of dietary diversity and speciated quickly upon arrival in the Nearctic and Neotropics. (PNG) [file pbio.3001414.s009.png]

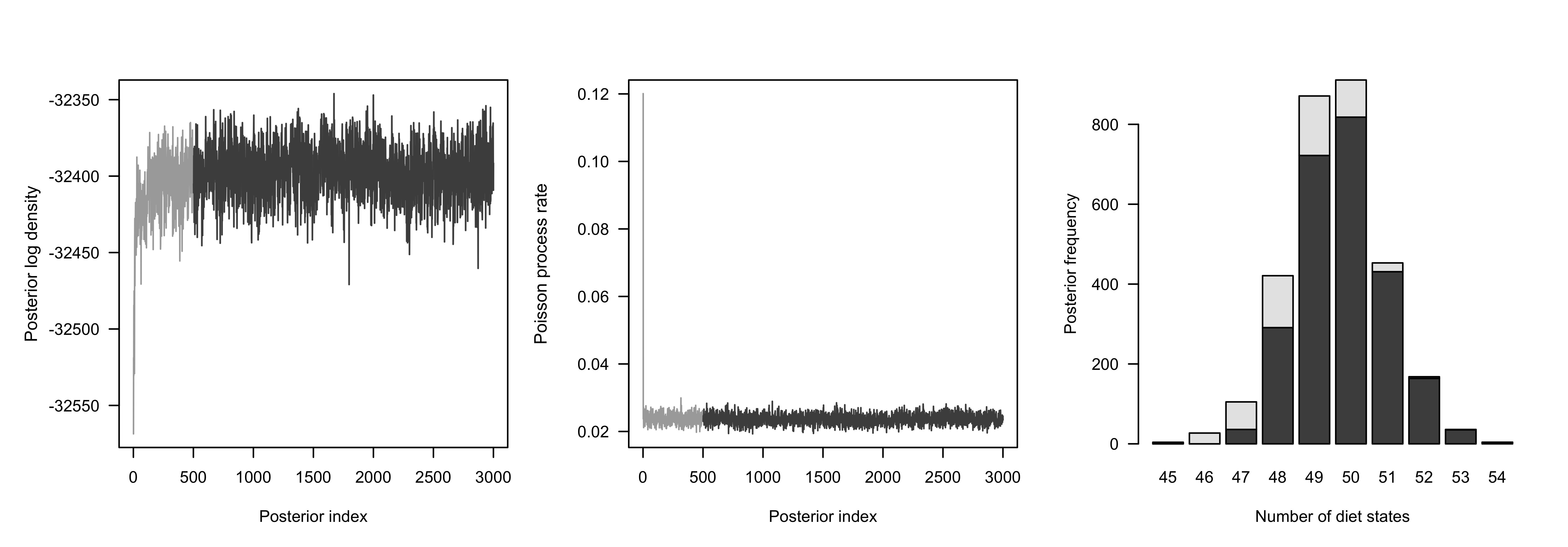

Supplement: S10 Fig — Likelihood (left) and parameter (middle) traces reveal good mixing of the Gibb’s sampler. The number of distinct dietary niches (right) sampled during the run is far less than the number of species (882), indicating that many sampled snake diets are indistinguishable from one another given the level of sampling. The highlighted portions correspond to the samples that were used to form posterior average summaries mentioned in the main text. (PNG) [file pbio.3001414.s010.png]

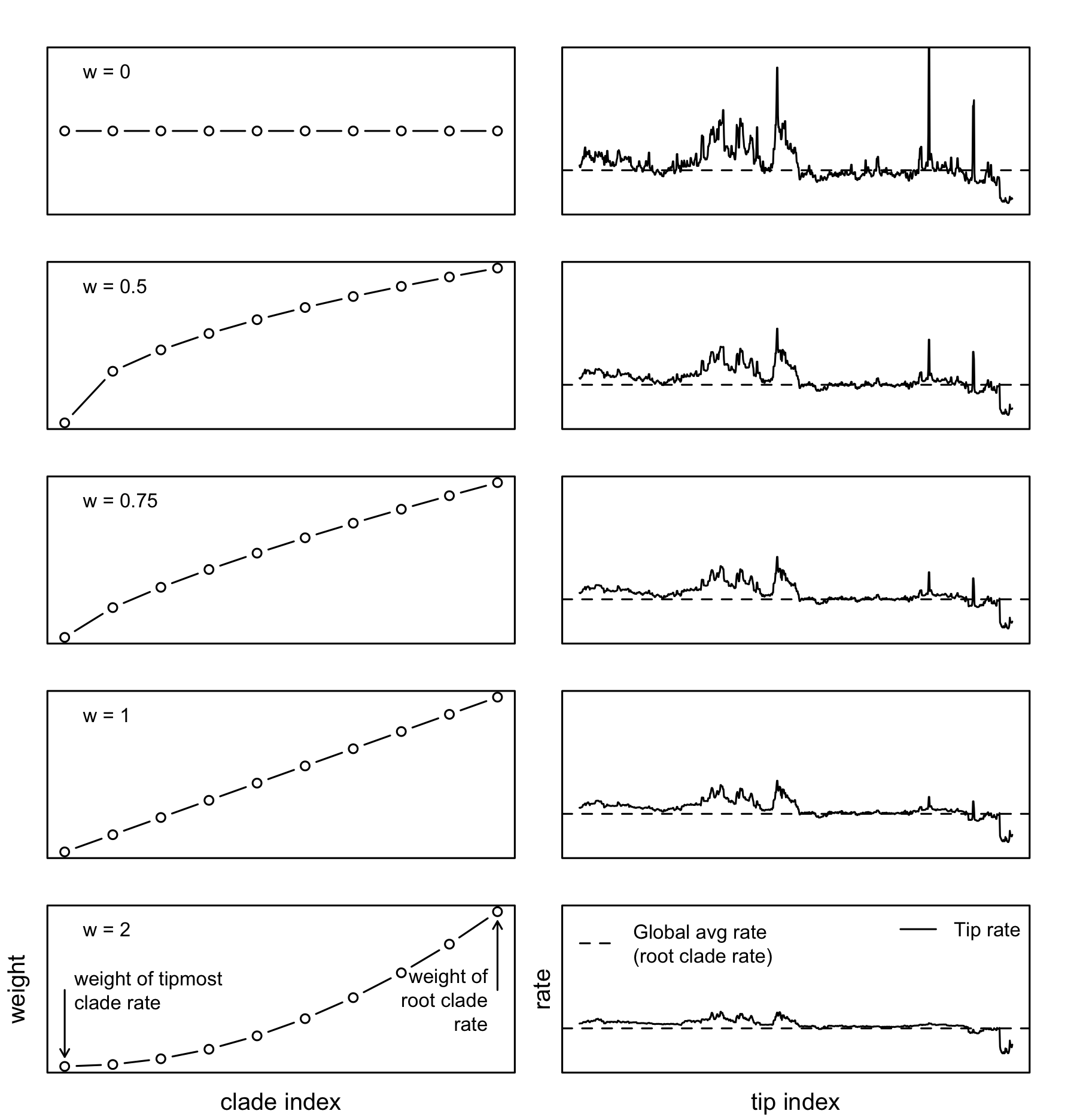

Supplement: S11 Fig — Different weighting schemes (left) applied to the clade rates shown in S9 Fig and their effect on tip rates (right). Tip rates are calculated as a weighted average of all clade rates on the phylogenetic path leading back to the root. While the weighting scheme influences the strength of relaxation toward the overall average rate, the qualitative pattern of tip rates is relatively unchanged by choice of weighting scheme. Tip rates in the main text (Fig 2) correspond to scheme w = 1. (PNG) [file pbio.3001414.s011.png]

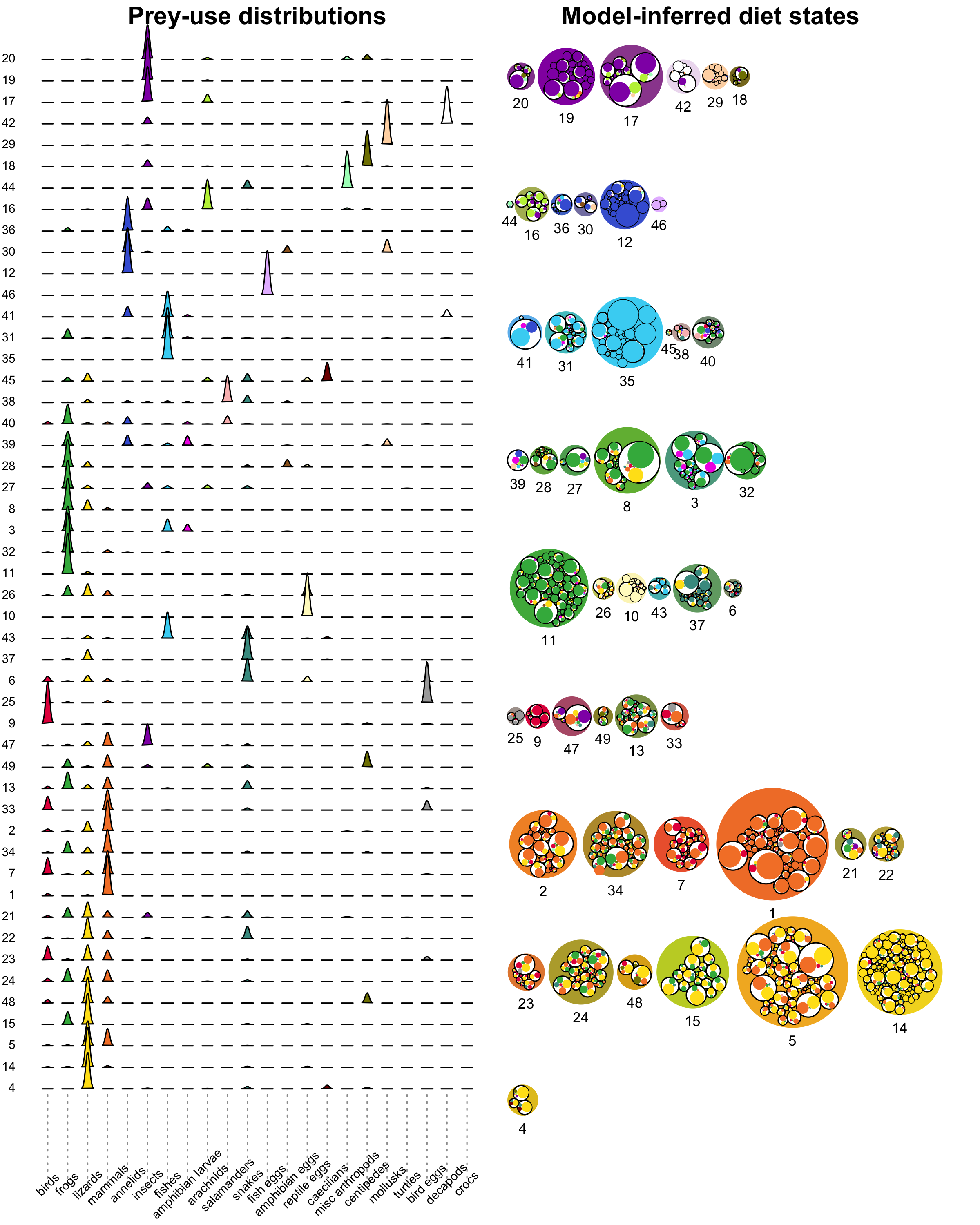

Supplement: S12 Fig — In the left column, each row represents the model-inferred prey use distribution underlying a particular diet state. In the right column, each circular cluster depicts the set of species assigned to a model-inferred diet state. Row numbers (left) and numbers inset below each cluster (right) correspond. Dietary states are identical to those shown in Fig 3. Circles outlined in black represent individual snake species, and subcircles within those circles represent different prey use observations, with circle size proportional to the number of observations. For example, state 12 (right panel; second row from top) represents an annelid specialist, with >0.95 of the multinomial distribution (left panel) concentrated on the annelid prey category. A total of 35 species were assigned to this state, visually represented by the outlined subcircles within state 12 (right panel); see main text Fig 1 for further interpretive information. In contrast, state 24 (left panel; sixth row from bottom) is a generalist, with approximately equal probabilities spread across frog, lizard, and mammal prey categories. (PNG) [file pbio.3001414.s012.png]

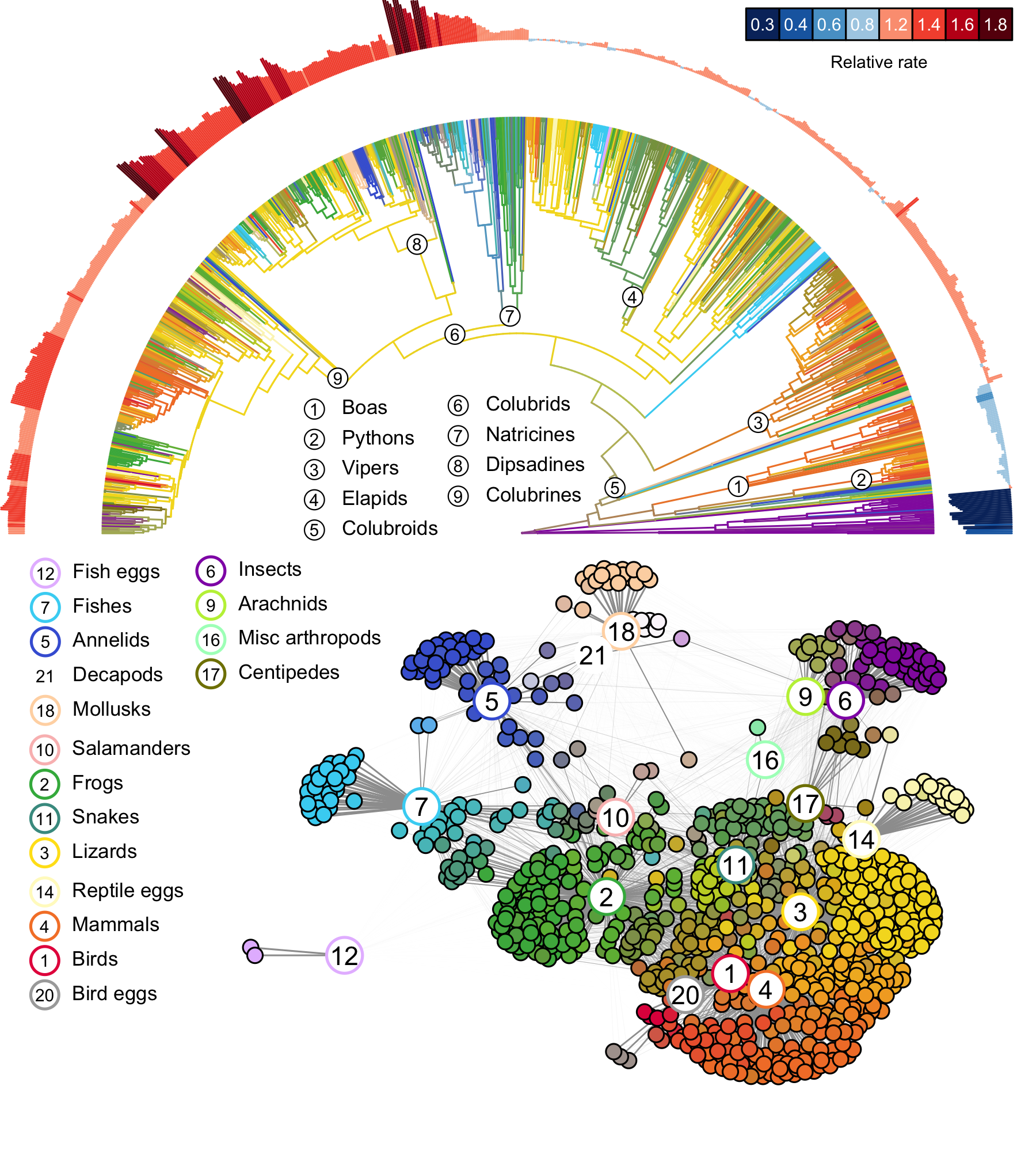

Supplement: S13 Fig — Note that the overall pattern is unchanged with the exception of muddier colored branches near the root, which reflects the greater uncertainty surrounding the diets of Mesozoic ancestors when K = 50 (cf S5 and S6 Figs). (PNG) [file pbio.3001414.s013.png]

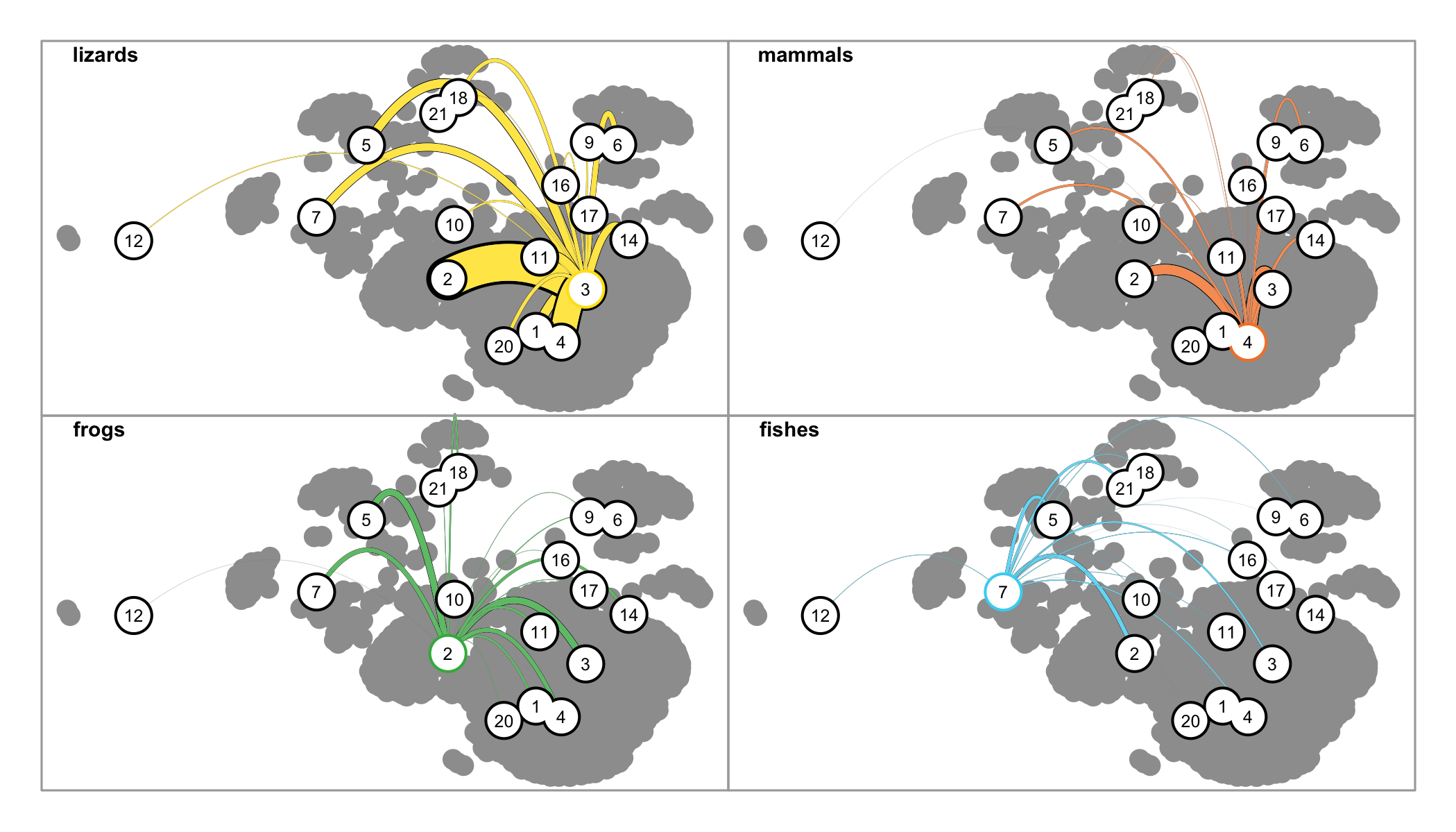

Supplement: S14 Fig — Note that greater uncertainty for Mesozoic ancestral states when K = 50 (cf S5 and S6 Figs) causes some posterior samples to favor early fish-eating ancestors, which increases the frequency of evolutionary transitions away fish diets relative to results in Fig 4 of the main text. (PNG) [file pbio.3001414.s014.png]
